# The Adoption of Artificial Intelligence in Health Care and Social Services in Australia: Findings From a Methodologically Innovative National Survey of Values and Attitudes (the AVA-AI Study)

**DOI:** 10.2196/37611

**Published:** 2022-08-22

**Authors:** Sebastian Isbanner, Pauline O’Shaughnessy, David Steel, Scarlet Wilcock, Stacy Carter

**Affiliations:** 1 Social Marketing @ Griffith Griffith Business School Griffith University Brisbane Australia; 2 School of Mathematics and Applied Statistics Faculty of Engineering and Information Sciences University of Wollongong Wollongong Australia; 3 Australian Research Council Centre of Excellence for Automated Decision-Making and Society The University of Sydney Law School The University of Sydney Sydney Australia; 4 Australian Centre for Health Engagement Evidence and Values Faculty of the Arts, Social Sciences and Humanities University of Wollongong Wollongong Australia

**Keywords:** artificial intelligence, surveys and questionnaires, consumer health informatics, social welfare, bioethics, social values

## Abstract

**Background:**

Artificial intelligence (AI) for use in health care and social services is rapidly developing, but this has significant ethical, legal, and social implications. Theoretical and conceptual research in AI ethics needs to be complemented with empirical research to understand the values and judgments of members of the public, who will be the ultimate recipients of AI-enabled services.

**Objective:**

The aim of the Australian Values and Attitudes on AI (AVA-AI) study was to assess and compare Australians’ general and particular judgments regarding the use of AI, compare Australians’ judgments regarding different health care and social service applications of AI, and determine the attributes of health care and social service AI systems that Australians consider most important.

**Methods:**

We conducted a survey of the Australian population using an innovative sampling and weighting methodology involving 2 sample components: one from an omnibus survey using a sample selected using scientific probability sampling methods and one from a nonprobability-sampled web-based panel. The web-based panel sample was calibrated to the omnibus survey sample using behavioral, lifestyle, and sociodemographic variables. Univariate and bivariate analyses were performed.

**Results:**

We included weighted responses from 1950 Australians in the web-based panel along with a further 2498 responses from the omnibus survey for a subset of questions. Both weighted samples were sociodemographically well spread. An estimated 60% of Australians support the development of AI in general but, in specific health care scenarios, this diminishes to between 27% and 43% and, for social service scenarios, between 31% and 39%. Although all ethical and social dimensions of AI presented were rated as important, accuracy was consistently the most important and reducing costs the least important. Speed was also consistently lower in importance. In total, 4 in 5 Australians valued continued human contact and discretion in service provision more than any speed, accuracy, or convenience that AI systems might provide.

**Conclusions:**

The ethical and social dimensions of AI systems matter to Australians. Most think AI systems should augment rather than replace humans in the provision of both health care and social services. Although expressing broad support for AI, people made finely tuned judgments about the acceptability of particular AI applications with different potential benefits and downsides. Further qualitative research is needed to understand the reasons underpinning these judgments. The participation of ethicists, social scientists, and the public can help guide AI development and implementation, particularly in sensitive and value-laden domains such as health care and social services.

## Introduction

### Background

Artificial intelligence (AI) and automation are accelerating in many fields driven by an increase in the availability of massive linked data sets, cloud computing, more powerful processors, and the development of new types of algorithms, particularly in the field of machine learning. In this paper, AI will be broadly conceptualized, consistent with the Australian Council of Learned Academies definition, as “a collection of interrelated technologies used to solve problems and perform tasks that, when humans do them, requires thinking” [1]. These technologies are being applied in social services, including to automate eligibility verification, target and personalize welfare services, and aid in the detection of fraud and debt liability [2,3]. Health care, initially slow to adopt AI, is also seeing rapid development for applications including health service planning and resource allocation, triage, screening and diagnosis, prognostication, robotics in applications such as aged care, and health advice chatbots [4-6]. These areas of practice—social services and health care—have traditionally been provided via extensive human-to-human contact by staff with professional autonomy and the capacity to exercise discretion in handling the problems of service users or patients.

### Ethical, Legal, and Social Implications of AI

A growing body of literature acknowledges the complex ethical, legal, and social implications (ELSI) of AI deployment [1,7,8]. In the 2010s, many intergovernmental, academic, and industry groups examined the ELSI of AI in a general sense, producing lists of high-level principles for AI ethics [1,7,9,10] often reminiscent of existing frameworks in bioethics [11]. In parallel, a set of approaches that foreground the significance of power, oppression, discrimination, and injustice has been developed, contextualized in the sociotechnical systems in which AI is embedded [12]. Other work critiques the corporate AI sector for establishing AI ethics boards and documents while persisting with unethical practices and points to the difficulties faced by AI ethics researchers when working inside corporations producing AI systems [13]. The abstract principles and frameworks that have proliferated in AI ethics offer accessible ways in to ethical debates, but they cannot be sufficient to address ethical issues in practice [14]. There are now calls to complement ethical frameworks with other forms of knowledge, including analysis of detailed use cases and investigation of what members of the public think and value regarding the use of AI [15]. Two linked cases are the focus of this study: the use of AI in health services and in social services, which are an important social determinant of health especially for marginalized and disadvantaged populations.

For the first case, health care AI, research on ELSI has been rapidly expanding since 2019. In a 2020 review, Morley et al [16] highlighted 3 groups of ELSI issues for health care AI: epistemic concerns (that the evidence on which health care AI is based is inconclusive, inscrutable, or misguided), normative concerns (highlighting unfairness and the potential for transformative unintended consequences), and concerns about the ability to either identify algorithmic harm or ascribe responsibility for it. Another 2020 review focused on health care emphasized the potential to worsen outcomes or cost-effectiveness, the problem of transportability (that algorithms may not work equally well in different populations), automation bias (that humans tend to be too willing to accept that algorithmic systems are correct), the potential to intensify inequities, the risk of clinical deskilling, increased threats to data protection and privacy, lack of contestability of algorithmic decisions, the need to preserve clinician and patient autonomy, and the potential to undermine trust in health care systems [17]. A 2021 scoping review on health care AI ELSI highlighted data privacy and security, trust in AI, accountability and responsibility, and bias as key ethical issues for health care AI [18]. Also in 2021, Goirand et al [19] identified 84 AI-specific ethics frameworks relevant to health and >11 principles recurring across these while noting that few frameworks had been implemented in practice. In parallel, empirical evidence demonstrates a continuing need to address the ELSI of health care AI. A well-known example is an AI system used to allocate health care in many US health services that allocated more care to White patients than to Black patients, even when the Black patients had greater need, because the AI learned from historical underservicing that Black patients had lower care requirements [20].

Regarding our second case, AI in the social services, ELSI research is also gaining momentum, particularly as part of broader inquiries into the digital welfare state or in relation to high-profile examples of technology failure [2,21,22]. This research highlights the potential of AI to improve the consistency and accuracy of welfare decision-making and increase cost-efficiency. However, it also raises grave concerns regarding the social costs associated with implementing AI in the social services, particularly for vulnerable populations. For example, the pioneering ethnographic study by Eubanks [21] of AI and automation technologies in the United States in 2018 illustrates how new technologies can disempower poor citizens, intensify existing patterns of discrimination, and *automate inequality*. Similar concerns have been raised in Australia in relation to the Online Compliance Intervention known as *robodebt*. The scheme automated the calculation of welfare debts based on an income-averaging algorithm. The legality of the algorithm was successfully challenged before a domestic court in 2019, culminating in an Aus $1.8 billion (US $1.25 billion) class action lawsuit against the Australian government and prompting significant public and scholarly criticism of the scheme [23].

AI applications in the welfare sector pose novel challenges to legal and regulatory compliance. Many AI systems, including robodebt, have been designed and implemented in the absence of proper legal frameworks or in contravention of prevailing laws and administrative principles [2,24]. Other high-profile examples include the System Risk Indication system of the Dutch government, which was used to predict an individual’s risk of welfare fraud. System Risk Indication was successfully challenged based on the fact that the system breached the right to privacy contained in the European Convention on Human Rights [2]. Such cases have prompted a growing body of literature concentrated on the legal and human rights implications of AI in the social services. The recent report by the United Nations Special Rapporteur on Extreme Poverty [2] calls for a human rights–based approach to digital regulation in social protection systems, which has prompted further research on AI and human rights principles [25].

### Existing Research on Perceptions of the ELSI of Using AI, Including in Health Care and Social Services

An approach to thinking about the ELSI of AI is to examine public attitudes and judgments toward these technologies. In areas such as health care and social services, this includes the attitudes and judgments of patients and service users. A small body of literature exists on general attitudes toward AI. In 2018, Zhang and Dafoe [26] surveyed 2000 American adults and found mixed support for developing AI and strong support for the idea that AI should be carefully managed. In April 2020, the Monash Data Futures Institute surveyed 2019 Australians on their attitudes toward AI, adapting some questions from Zhang and Dafoe [27]. They found that Australians did not consider themselves knowledgeable about AI, but 62.4% expressed support for the development of AI. When asked whether they supported the use of AI in particular fields, respondents were most supportive of AI use in health (44.1% strong support) and medicine (43% strong support) and less supportive of AI use in *equality and inclusion* (21.5% strong support) and public and social sector management (20.2% strong support). Respondents tended to agree that AI would do more social good than harm overall [27].

Research on the attitudes of patients and service users is developing; most research to date—such as this study—has been speculative, asking informants about their views or intentions rather than their direct experience of AI. Studies asking patients to imagine the use of AI in their care generally report broad acceptance [28-30] in areas including skin cancer screening and neurosurgery. Promises of greater diagnostic accuracy are well received [30], and sharing deidentified health data for the development of medical AI may be acceptable to most [28]. A study reported experiences with a diabetic retinopathy screening AI—96% of patients were satisfied or very satisfied [31]. However, respondents in most studies also express concerns. Regarding skin cancer screening, concerns included inaccurate or limited training sets; lack of context; lack of physical examination; operator dependence; data protection; and potential errors, including false negatives and false positives [28,30]. In the context of neurosurgery, respondents wanted a human neurosurgeon to remain in control [29]. Finally, a study of patients with cancer in China suggested that despite reporting that they *believed in* both diagnoses and therapeutic advice given by an AI (90% and 85%, respectively), when this differed from the advice given by a human clinician, most patients would prefer to take the human clinician’s recommendation (88% and 91%, respectively) [32].

Research examining public and professional attitudes toward AI in the welfare sector is very limited. To the authors’ knowledge, research is yet to explore citizens’ general attitudes toward AI in the domain of welfare provision. However, there is a small body of research documenting service users’ experiences of specific AI applications in the social services, particularly users’ negative experiences of exclusion and discrimination [21,33], providing context-specific insights into system users’ experiences of AI and illustrating the high-stakes nature of implementing AI in this domain. This work, together with some small-scale, mostly qualitative studies involving frontline social service staff [34-38], illustrates the complex and dynamic relationship between AI and the routines of social welfare professionals and indicates mixed reactions to these systems among staff. For example, the study by Zejnilović et al [36] of counselors in a Portuguese employment service in 2020 found high levels of distrust and generally negative perceptions of an AI system used to score clients’ risk of long-term unemployment. However, the survey data also indicated that workers would continue to rely on the system even if it became optional, suggesting that respondents harbor mixed feelings about the system.

The Australian Values and Attitudes on Artificial Intelligence (AVA-AI) study set out to understand Australians’ values and attitudes regarding the use of AI in health care and social services. Australia has been relatively slow to approve and adopt medical AI compared, for example, with the United Kingdom and the United States. The adoption of AI and automation technologies in the social services is comparatively advanced in Australia, although its development has been uneven and marked by controversy, including the case of robodebt. Multiple stakeholders are now confronting the opportunities and risks of these technologies. Policy makers need high-quality evidence of what Australians consider acceptable or unacceptable to ensure that their decision-making is legitimate. This study used an innovative methodology to survey Australians regarding these questions. Our aims were to understand Australians’ front-of-mind normative judgments about the use of AI, especially in the underresearched fields of social services and health care, and what attributes of AIs they would consider to be most important if those AIs were to be deployed in health care and social services. Although parallel literature seeks to model the characteristics of AI that predict acceptance [39], this work has the complementary aim of seeking to understand the prevalence and patterning of different normative judgments about AI.

The research questions answered in this study are as follows: (1) How do Australians’ general judgments regarding the use of AI compare with their judgments regarding the particular uses of AI in health care and social services? (2) Do Australians make different judgments about different health care and social service applications of AI? (3) What attributes of health care and social service AI systems do Australians consider most important?

## Methods

### Aims

The *AVA-AI study* was conducted to (1) provide information on Australians’ attitudes and values regarding AI, especially in health care and social services, and (2) allow for analysis of how these vary across different subpopulations and are associated with people’s sociodemographic characteristics and familiarity with technology. This study focuses on attitudes and values, how they differ for different scenarios, and the relative importance of different attributes of health care and social service AI. A selection of concepts from AI ethics relevant to understanding this study is outlined in Textbox 1. Analyses across different subpopulations will be reported in future papers.

**Concepts from artificial intelligence (AI) ethics used in the Australian Values and Attitudes on Artificial Intelligence (AVA-AI) study.**

**Concept and meaning in the context of AI ethics**

- Accuracy: the degree to which an AI can perform tasks without errors. In the context of screening or targeting, for example, this would include the ability of the AI to detect a condition or identify a person without false positives (where a case is identified as having a condition or being a target when they do not fit the criteria). It also includes the ability of the AI to avoid false negatives (where a case is identified as not having a condition or not being a target when they do fit the criteria).
- Algorithmic targeting: the use of AI to find people with a certain profile, often predictively (eg, to identify people likely to be unable to find work or people likely to commit a crime).
- Autonomous machine decision-making: situations in which an AI makes a decision that would previously have been made only by a person, for example, whether a person has a condition or whether a person is eligible for a social security payment.
- Contestability: whether machine decision-making can be effectively challenged. Contestability is to some extent dependent on explainability but is also dependent on policy settings.
- Explainability: whether it is possible to explain how an AI makes a decision. For some forms of AI, especially deep learning algorithms, humans do not explicitly instruct the AI on what basis it should make decisions. This makes explainability potentially more challenging, leading such algorithms to be labeled as *black box* algorithms.
- Deskilling: when tasks previously undertaken by humans are delegated to AI, humans lose their ability to complete those tasks; that is, they deskill in relation to those tasks.
- Fair treatment: AI systems tend to reflect human bias; this relates to the concept of justice, which is complex and multidimensional. Doing justice is unlikely to entail treating everyone identically as different people have different needs and opportunities. In the AVA-AI study, we asked respondents how important it was to “know that the system treats everyone fairly” to capture an intuitive judgment of a system’s capacity to deal justly or unjustly with different individuals and populations.
- Personal tailoring: the ability of an AI, by comparing the data of an individual with large, linked data sets, to recommend services or interventions that respond to the particularity of an individual’s situation.
- Privacy: freedom from intrusion into personal matters, including the ability to control personal information about oneself.
- Responsibility: a complex and multidimensional concept, which attributes moral or legal duties and moral or legal blame, including for errors or harms.

### Instrument Development

When designing the study, there were no existing instruments we could adopt. We used a question from the 2018 survey by Zhang and Dafoe [26] and developed other questions based on a review of the AI ethics literature. Before the study commenced, the instrument underwent multiple rounds of input from investigators and expert colleagues, as well as cognitive testing.

### Final Instrument Design

In addition to sociodemographic variables, the survey asked about the use of AI in health care and welfare. Questions were of 2 types. The first type, in the form of *How much* *do you support or oppose,* presented a 5-point scale. Questions of this type asked about the development of AI in general (B01, taken from Zhang and Dafoe [26], running from *strongly support* to *strongly oppose*) and the use of AI in 6 particular health care and welfare AI scenarios for which potential advantages and disadvantages were presented in a balanced way (C03-C05 and D03-D05, for which the 5-point scale ran from *I support this use of AI* to *I oppose this use of AI*; Multimedia Appendix 1). A final question of this type (E01) asked respondents to indicate what they valued more on a 5-point scale: *Quicker, more convenient, more accurate health and social services* or *More human contact and discretion in health and social services.* This trade-off asked respondents to evaluate a bundle of benefits commonly attributed to AI-enabled services against a bundle of benefits commonly attributed to services provided by human professionals.

The second type of question presented a scenario involving AI use and then asked respondents to consider 7 ELSI dimensions or values (eg, *getting an answer quickly* and *getting an accurate answer*) and rate how important each dimension was to them personally on a scale from *extremely important* to *not at all important*. There were 4 questions of this type: 2 with health care scenarios (C01-C02) and 2 with welfare scenarios (D01-D02). Module C presented health care questions and module D presented welfare questions; respondents were randomly allocated to receive module C or D first, and the order of presentation of the values was also randomized. Table 1 summarizes the variables presented as well as the concepts each question was designed to assess. Note that the dimensions or values were identical for module C and D questions except that the health care questions had an item about responsibility, including mistakes (reflecting the status quo of medical professional autonomy), whereas the social service questions had an item about personal tailoring (reflecting a promised potential benefit of AI in social services).

The final survey instrument is provided in Multimedia Appendix 1.

**Table 1:** Summary of the variables collected in the Australian Values and Attitudes on Artificial Intelligence (AI) study.

Type of variable	Question number and variable	Concepts tested
General support or opposition	B01—how much do you support or oppose the development of AI in general (with multiple examples given)?^a^	Broad support for or opposition to AI
Importance of different attributes of AI in health care scenarios	C01—machine reads medical test, diagnoses, and recommends treatment C02—machine triages when you are unwell	In relation to: C01—delegation of clinical decisions to an autonomous machine C02—automating decisions about need for health care services (time-sensitive) Importance of: Explanation Speed Accuracy Human contact Reducing system costs Fair treatment Responsibility
Importance of different attributes of AI in welfare scenarios	D01—machine processes application for unemployment benefits (data sharing required) D02—chatbot advises about carer payments	In relation to: D01—foregoing privacy as a barrier to access services D02—automation of information services Importance of: Explanation Speed Accuracy Human contact Reducing system costs Fair treatment Personal tailoring
Support for or opposition to AI in specific health care scenarios	C03—nonexplainable hospital algorithms C04—data sharing for quality care C05—deskilling physicians	C03—importance of explainable machine recommendations C04—importance of privacy (balanced against quality of care) C05—importance of retaining human clinical skills
Support for or opposition to AI in specific welfare scenarios	D03—targeted compliance checking D04—nonexplainable job services D05—automated assignment of parent support with limited contestability	D03—algorithmic targeting of punitive policy D04—importance of explainable machine recommendations D05—importance of contestability (balanced against accuracy)
Speed—human contact	E01—trade-off between quicker, more convenient, more accurate health care and social services and more human contact and discretion in health care and social services	E01—speed and convenience and accuracy vs human contact and discretion
Sociodemographic	Age, gender, concession card type, and employment status; household income, education, household type, language other than English spoken at home, and general health Centrelink payment, employment field, relevant experience, relevant degree, life satisfaction, and disability	Descriptive variables collected using standard sociodemographic questions
Geographic	State or territory, capital city or rest of state, and SEIFA^b^ (geographic measure of disadvantage)	Descriptive variables collected using standard questions about location of residence
Lifestyle	How often they check the internet, how often they post comments or images to social media, how often they post on blogs, forums, or interest groups, early adopter by type, and television viewing by type of viewing	Variables collected for weighting purposes

^a^Variables in italics were collected from both the Life in Australia and web-based panel samples; all others were collected from the web-based panel alone.

^b^SEIFA: Socio-Economic Indexes for Areas.

### Data Collection Processes and Weighting

Data collection occurred between March 16, 2020, and March 29, 2020, with respondents mainly completing the questionnaire on the web.

The AVA-AI study comprises 2 sample components: one obtained from the Life in Australia (LIA) survey [40] with a responding sample size of 2448 and a web-based panel sample with a responding sample size of 2000. Thus, the combined responding sample size was 4448.

The full set of questions was used for the web-based panel sample. For the LIA sample, a subset of sociodemographic variables and all the geographic and lifestyle questions were used. The LIA sample also answered the general support question (B01) and the importance of AI attributes for scenario C01. In Table 1, the variables in italics were collected from both the LIA and web-based panel samples, and all others were collected from the web-based panel alone.

The LIA sample was selected using scientific probability sampling methods, whereas the web-based panel sample was a nonprobability sample. Weights for the LIA sample were calculated using standard methods for a probability sample using generalized regression estimation [41] to adjust for differences in selection probabilities and nonresponse and calibrate to population benchmarks obtained from the population census, current demographic statistics, and the 2017 to 2018 National Health Survey obtained from the Australian Bureau of Statistics. The variables used in the calibration were age by highest education level, country of birth by state, smoking status by state, gender by state, household structure by state, part of state, and state or territory.

A web-based panel allowed us to generate a relatively large sample, enabling a good level of disaggregation into subpopulations, comparisons between groups, and analysis of associations. Such panels can be subject to self-selection biases and coverage issues, reducing the accuracy of population prevalence estimates [42], but may enable the examination of associations and, with adjustments to reduce biases, improve the estimation of population characteristics [43]. The calibration to population benchmarks for major sociodemographic variables may not eliminate these issues. To enhance our adjustment of the web-based panel data in the AVA-AI study, we included 2 substantive questions, a set of behavioral and lifestyle questions, and major sociodemographic variables in both the web-based panel survey and the probability sample–based LIA survey, as indicated in Table 1. This approach was similar to that used in the study by Zhang and Dafoe [26], although our approach for the AVA-AI study went further by adjusting for behavioral and lifestyle variables and 2 substantive variables. The use of behavioral and lifestyle variables in adjusting web surveys, also known as webographic variables, is discussed in the study by Schonlau et al [44], for example.

In the AVA-AI study, questions common to the LIA and web-based panel samples were used to calibrate the web-based panel to the LIA sample, producing weights designed to reduce potential biases owing to the web-based panel sample being nonrandom; the LIA served as a reference survey [35]. The probability of inclusion for the web-based panel respondents was estimated using a propensity score model. This involved combining the LIA and web-based panel samples and fitting a logistic regression model, with the response variable being membership of the web-based panel. In fitting this model, the original LIA weights were used for respondents in that sample, and a weight of 1 was used for the web-based panel respondents. The variables used in the logistic regression were selected using Akaike Information Criterion–based stepwise regression and consisted of age by education, gender, household structure, language spoken at home, self-rated health, early adopter status, and television streaming watching. In a final calibration step, the weights were further adjusted to agree with the population benchmarks for these variables. This approach is described, for example, in the study by Valliant and Dever [45,46] and by Elliot and Valliant [47]. The weighting led to a weighted sample of 1950 for the web-based panel and 2498 for the LIA sample.

### Statistical Analysis Methods

#### Overview

All estimates and analyses were based on a weighted analysis using the largest sample possible. Each respondent had a weight determined by the sample they came from. The weights were scaled so that the sum of the weights for the combined sample was 4448. Two substantive questions (B01 [general support or opposition] and C01 [support or opposition for autonomous machine decision-making in medical testing]) were asked to the combined LIA+web-based panel sample. The remainder of the attitude and value questions was asked only to the web-based panel sample. Any analysis involving questions included in the LIA and web-based panel sample was based on the combined sample and the associated weights. Any analysis involving questions that were only collected from the web-based panel sample was based on the web-based panel sample and the associated weights.

The analyses focused on determining and comparing the distribution of responses to the attitude and value questions. The methods used accounted for the use of weights in calculating estimates and associated 95% CIs and allowed for the testing of statistical significance, assessed when the *P* value of the relevant statistical test was <.05.

#### Statistical Analysis of Each Question Using Univariate Analyses

All variables concerning attitudes and values had 5 substantive response categories reflecting *support* or *importance.* Univariate analysis calculated the estimated percentage in each response category for each question, with 95% CIs for each estimated percentage. For questions asking for degree of support or opposition, we examined whether there was a majority support and compared across scenarios and between health care and welfare contexts; for questions asking for the importance attached to different attributes or values, we examined whether attributes or values mattered more in some contexts than others.

Weights must be accounted for in the calculation of estimates and in the statistical inference, such as estimates of SEs and the associated CIs obtained from them and *P* values for any statistical tests used. The CIs and *P* values were obtained using *Complex Samples* in SPSS (version 26; IBM Corp), which accounts for the use of weights in producing the estimates. Although the use of weights can reduce bias, there is an associated increase in variances and SEs of the estimates. This is reflected in the design effect, the variance of an estimate accounting for the weights (and complex design if used), compared with the use of simple random sampling and no weighting. The effect is variable specific, but a broad indication can be obtained considering the design effect because of weighting or unequal weighting effect [48,49]. This is 1+*Cw2*, where *Cw* is the coefficient of variation of the weights, which is the SD of the weights divided by their mean. For the combined sample, the design effect because of weighting was 1.83; for the LIA, it was 1.99; and, for the web-based panel, it was 1.61. For any specific estimates or analysis in this study, the SEs estimated from the survey data accounting for the weights were used. The effect on the SE is the square root of the design effect (ie, the design factor [50]) and is the factor by which the CIs are larger than if weights did not have to be used. A design effect of 1.83 implies a design factor of 1.35. In this analysis, the design effects were almost all between 1.50 and 2.00.

For questions using ordinal scales from 1 to 5, we also calculated an overall mean response to each question and the associated 95% CI. These included variables assessing the degree of support (ie, B01, C03-C05, and D03-D05), importance attached to attributes of AI (ie, C01-C02 and D01-D02), and the final question (E01) on trading off machine versus human traits. Mean scores close to the midpoint of the scale (3.00) indicated an overall neutral or balanced response to the question, that is, an equal or symmetric distribution of respondents on the respective scale. For support-or-oppose questions, lower scores indicated support and higher scores indicated opposition; for importance questions, lower scores indicated greater importance and higher scores indicated less importance; for E01, lower scores favored machine traits and higher scores favored human traits. For all questions, we tested the null hypothesis that the mean was 3.00 (ie, a distribution centered at the midpoint of the scale, or a balanced distribution of responses) using a 2-tailed *t* test allowing for weighting.

#### Statistical Analysis Comparing Responses to Questions Using Bivariate Analyses

To assess differences in the responses to pairs of questions—for example, is the support for the use of AI different when respondents are presented with different scenarios?—we compared the distributions of the responses. This was not to assess whether the responses to the 2 questions were independent, which is unlikely, but whether the percentages in their marginal distributions were the same.

Our goal was to determine what percentage of people changed their response between 2 questions and whether this change was net positive or negative. To examine this issue for any 2 questions, we created a *shift variable* to represent the difference between two variables (variables A and B): (1) if the response to variable A was in a category greater than the response to variable B, the *shift variable* was +1, which corresponded to a more positive attitude toward AI for variable B and, equivalently, a more negative attitude for variable A; (2) if the response to variable B was in a category greater than the response to variable A, the *shift variable* was −1, which corresponded to a more positive attitude toward AI for variable A and, equivalently, a more negative attitude for variable B; and (3) if the responses to variables A and B were identical, the *shift variable* was 0.

We estimated the percentage of respondents where the *shift variable* was 0, indicating no change. For those that changed, we estimated the percentage with a shift variable of −1, corresponding to a more positive attitude for the first variable and a more negative attitude for the second variable, and tested for equal percentages of positive and negative changes. The adjusted Pearson chi-square test in SPSS *Complex Samples* was used, which is a variant of the second-order adjustment proposed by Rao and Scott [51]. These tests allowed us to assess the statistical significance of the differences in responses under different scenarios.

We also tested for equal marginal distributions using the ordinal scores. SPSS uses a paired *t* test using these scores, which is similar to the test for marginal homogeneity described in the study by Agresti [52]. This test was implemented accounting for the weights using *Complex Samples* in SPSS by creating a variable for each person equal to the difference between the scores of the 2 questions and testing that the mean difference was 0. We tested answers to our research questions, that is, to determine whether respondents answered differently when questions tested the same ELSI concept in different settings or when questions tested different ELSI concepts in comparable settings. The estimated mean difference and associated 95% CI and the *P* value for the test that the mean difference was 0 were produced.

### Ethical Considerations

This study was approved by the University of Wollongong Social Sciences Human Research Ethics Committee (protocol number 2019/458).

## Results

### Sample Composition

Table 2 provides a summary of the weighted combined sample and web-based panel sample for the key variables. A full composition of the overall combined sample and the web-based panel, including unweighted and weighted frequencies and proportions for key sociodemographic variables, is provided in Multimedia Appendix 2. The use of weights improved the representation of the combined sample for capital cities, age groups <35 years, men, employed status, nonuniversity as the highest level of education, language other than English spoken at home, those with excellent or very good health, and people who look for information over the internet several times a day. The sample was well spread and had respondents across many different sociodemographic groups.

The web-based panel sample was also well spread across many different sociodemographic groups. The effect of weighting was similar to that in the overall sample, although there was very little effect for age and capital cities. Comparing the weighted percentages between the combined sample and the web-based panel sample, the only appreciable difference is for those employed (2709/4448, 60.9% vs 1061/1950, 54.41%, respectively).

**Table 2:** Sociodemographic composition of Australian artificial intelligence survey sample (weighted data only).

		Combined sample (n=4448), n (%)	Web-based panel (n=1950), n (%)
**Part of state**			
	Capital city	2957 (66.48)	1300 (66.67)
	Rest of state	1481 (33.3)	640 (32.82)
	Not stated or unknown	10 (0.22)	10 (0.51)
**Age group (years)**			
	18 to 34	1386 (31.16)	637 (32.67)
	35 to 54	1472 (33.09)	660 (33.85)
	55 to 74	1166 (26.21)	497 (25.49)
	≥75	394 (8.86)	156 (8)
	Not stated or unknown	30 (0.67)	0 (0)
**Gender**			
	Men	2180 (49.01)	939 (48.15)
	Women	2259 (50.79)	1011 (51.85)
	Other	9 (0.2)	1 (0.05)
	Not stated or unknown	0 (0)	0 (0)
**Employment status**			
	Employed	2709 (60.9)	1061 (54.41)
	Not employed	1735 (39.01)	890 (45.64)
	Not stated or unknown	4 (0.09)	0 (0)
**Highest education level**			
	Postgraduate qualification	529 (11.89)	246 (12.62)
	Undergraduate or diploma	1393 (31.32)	676 (34.67)
	Vocational qualification	937 (21.07)	398 (20.41)
	School qualification	1492 (33.54)	626 (32.1)
	Not stated or unknown	96 (2.16)	5 (0.26)
**Gross weekly household income**			
	≥Aus $3000 (US $2086.20)	635 (14.28)	211 (10.82)
	Aus $1500 to Aus $2999 (US $1043.10 to US $2085.50)	1281 (28.8)	589 (30.21)
	Aus $500 to Aus $1499 (US $347.70 to US $1042.40)	1646 (37.01)	793 (40.67)
	<Aus $500 (US $347.70)	550 (12.37)	261 (13.38)
	None	139 (3.13)	70 (3.59)
	Negative income	34 (0.76)	26 (1.33)
	Not stated or unknown	162 (3.64)	0 (0)
**Other language spoken at home**			
	Yes	1036 (23.29)	438 (22.46)
	No	3411 (76.69)	1513 (77.59)
	Not stated or unknown	1 (0.02)	0 (0)
**General health**			
	Excellent	549 (12.34)	236 (12.1)
	Very good	1887 (42.42)	837 (42.92)
	Good	1302 (29.27)	562 (28.82)
	Fair	573 (12.88)	255 (13.08)
	Poor	131 (2.95)	59 (3.03)
	Not stated or unknown	6 (0.13)	0 (0)

### Support for AI in General and in Specific Scenarios

#### Background

We first discuss questions focused on support for or opposition to AI. The CIs for questions B01 and C01 tended to be narrower as they were based on the combined sample. However, for all questions, estimates of percentages had margins of error (ie, twice the SE) of <3 percentage points, reflecting the relatively large sample size and the reliability of all estimates.

#### Respondents Expressed General Support for AI

**Figure 1:**
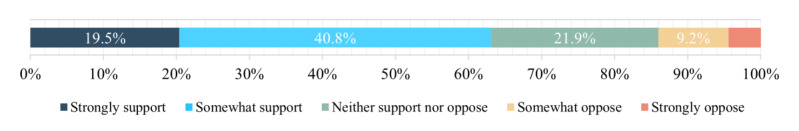
Responses to question B01: How much do you support or oppose the development of artificial intelligence?

Figure 1 and Table 3 show the level of support for the development of AI in general—an estimated 60.3% in the *strongly support* or *somewhat support* categories.

Although the estimate for the *support* categories was 60.3%, it was only 13.4% for the *opposed* categories and 26.3% for the *neutral* or *don’t know* responses. The on-balance support mean score of 2.35 was statistically significant when tested against the midpoint of 3.00 (*P*<.001). The design effects are consistent with the design effect that was due to a weighting of 1.83.

Table 4 shows the percentage that selected a *support* category after *don’t know* responses were excluded and also after don’t know and neutral responses were excluded. This allowed for direct comparison of support and opposition and examination of whether there was majority support. We tested whether the resulting percentages were >50% using the adjusted Pearson F test for equal percentages in SPSS, where an estimate of 50% would indicate equal levels of support and opposition. Table 4 clearly demonstrates majority support among those taking a positive or negative position—63.1% when *don’t know* responses were excluded and 81.8% when neutral and don’t know responses were excluded, with *P* values indicating that both estimates were statistically significantly different from 50%.

For each question in the remaining analyses, the very small proportion of refused and *don’t know* responses were not included and were no more than 8 cases for any of these questions.

**Table 3:** Estimated percentages, mean, and 95% CIs for responses to question B01: How much do you support or oppose the development of artificial intelligence?^a,b^

	Estimated percentage (95% CI)	Design effect
Strongly support	19.5 (17.9-21.1)	1.87
Somewhat support	40.8 (38.9-42.8)	1.84
Neither support nor oppose	21.9 (20.3-23.5)	1.74
Somewhat oppose	9.2 (8.1-10.4)	1.87
Strongly oppose	4.2 (3.5-5.1)	1.76
I don’t know	4.4 (3.6-5.3)	1.96

^a^Percentages and CIs adjusted for weighting.

^b^The mean score was 2.35 (95% CI 2.31-2.39) with a design effect of 1.83.

**Table 4:** Percentage of those who strongly support or somewhat support the development of artificial intelligence, 95% CIs, and *P* values for testing against 50%^a^.

	Categories deleted	
	“Don’t know”	“Don’t know and neutral”
Estimated percentage support (95% CI)	63.1 (61.1-65)	81.8 (80-83.5)
*P* value^b^	<.001	<.001
Design effect	1.80	1.83

^a^Percentages and CIs adjusted for weighting.

^b^*P* value for adjusted Pearson *F* test for equal proportions in *support* and *oppose* categories.

#### Respondents Showed Less Support for Specific AI Use Scenarios and Supported Some Scenarios More Than Others

**Figure 2:**
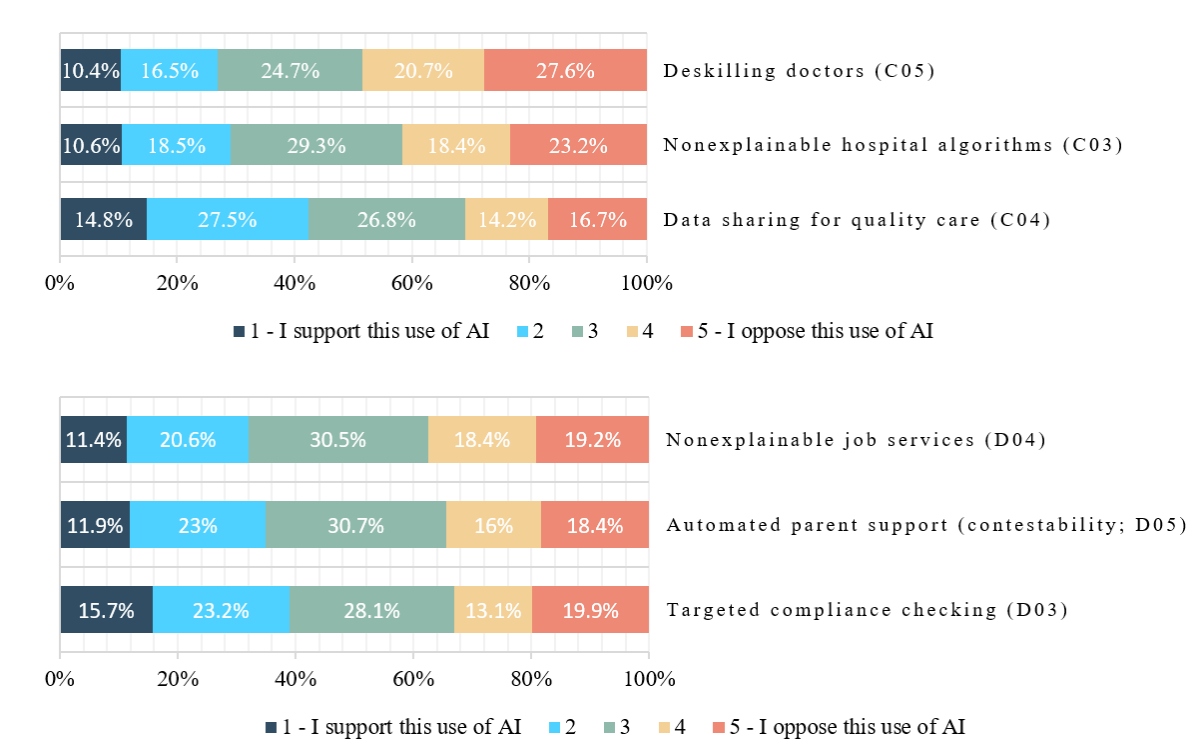
Responses to questions C03 to C05 and D03 to D05: support for or opposition to specific scenarios. AI: artificial intelligence.

Figure 2 shows the estimates of the level of support for AI in specific health care and welfare scenarios, with scenarios presented in increasing order of level of support. Multimedia Appendix 3 shows the related estimates and 95% CIs. Table 5 presents estimates of support in categories 1 and 2 combined for specific scenarios, associated 95% CIs, and *P* values for the test against 50%. For all these specific scenarios, less support was expressed than in the question about AI in general (Figure 1).

Figure 2 shows that the strongest support was expressed for a learning health care system making diagnostic and treatment recommendations, where *over time, patients get different care depending on whether they do, or do not, share their health record with the AI system* (ie, people receive health benefits only at the expense of health data privacy). Overall, the support for this item was 42.3% (Table 5). Regarding social services, the highest level of support was for targeted compliance checking for welfare debt (38.9%). In this scenario, a government department used an algorithm to check groups deemed *high-risk* for welfare overpayment twice as often, which found more welfare debts, saved money, and reduced the number of checks on other people but meant people in high-risk groups were checked more even if they had not done anything wrong. The next highest support was for automated systems to identify parents who required assistance to return to work with limited contestability (34.9%) and employment support recommendation systems that were nonexplainable to employment service workers (31.2%). The least support overall was expressed for AI systems that led to physician deskilling (27% support and 48.3% opposition) and those that made diagnostic and treatment recommendations but were not explainable to physicians (29.1% support and 41.6% opposition).

For the estimates in Table 5, the neutral middle category with a score of 3 was included in the denominator. To directly compare the level of support and opposition and assess whether there was majority support or opposition, we removed the neutral category and recalculated the estimates and tests (Table 6). With the neutral score included, the level of support never reached a majority and ranged from 27% (deskilling physicians) to 42.3% (data sharing for quality care). Once the middle category was excluded, Table 6 shows that, for the nonneutral respondents, there were majorities supporting data sharing and targeted compliance checking; a balance on automated parent support without contestability; and a majority opposed to nonexplainable hospital algorithms, nonexplainable job services, and especially deskilling physicians.

Table 7 uses mean scores to indicate on-balance opposition or support—a score >3.00 indicates on-balance opposition, and a score <3.00 indicates on-balance support, along with *P* values for testing that the mean score was 3 (neither supportive nor opposed on balance). The means of general support for the development of AI were included for comparison. Marginal on-balance support was demonstrated for data sharing for quality care only (this should not be overinterpreted as the mean score was so close to neutral). For targeted compliance checking and noncontestable automated parent support, views were balanced. For both explainability scenarios and clinical deskilling, respondents expressed on-balance opposition at a statistically significant level.

**Table 5:** Percentage of those supporting artificial intelligence in specific scenarios, 95% CIs, and *P* values for testing against 50%^a^.

Domain and scenario			Estimated percentage in “support” or “strongly support” categories (95% CI)		*P* value^b^			Design effect
**Health**								
	Data sharing for quality care (C04^c^)	42.3 (39.6-45.1)		<.001			1.62	
	Nonexplainable hospital algorithms (C03)	29.1 (26.7-31.6)		<.001			1.57	
	Deskilling physicians (C05)	27 (24.6-29.5)		<.001			1.57	
**Welfare**								
	Targeted compliance checking (D03)	38.9 (36.2-41.7)		<.001			1.61	
	Automated parent support (contestability; D05)	34.9 (32.3-37.6)		<.001			1.59	
	Nonexplainable job services (D04)	31.2 (28.7-33.8)		<.001			1.56	

^a^Percentages and CIs adjusted for weighting.

^b^*P* value for adjusted Pearson *F* test for 50% proportions in categories 1 and 2 combined.

^c^Code in parentheses (eg, C04) indicates question number in instrument.

**Table 6:** Proportion of respondents supporting artificial intelligence in specific scenarios, associated 95% CIs, and *P* values for testing against 50%; neutral responses deleted^a^.

Domain and scenario		Estimated percentage in “support” or “strongly support” categories	*P* value^b^		Design effect
**Health**					
	Data sharing for quality care (C04^c^)	57.8 (54.5-61.1)	<.001		1.63
	Nonexplainable hospital algorithms (C03)	41.1 (38-44.4)	<.001		1.58
	Deskilling physicians (C05)	35.8 (32.8-38.9)	<.001		1.58
**Welfare**					
	Targeted compliance checking (D03)	54.1 (50.9-57.4)	.01		1.58
	Automated parent support (contestability; D05)	50.4 (47-53.7)	.82		1.62
	Nonexplainable job services (D04)	44.1 (40.8-47.4)	<.001		1.59

^a^Percentages and CIs adjusted for weighting.

^b^*P* value for adjusted Pearson *F* test for 50% proportions in categories 1 and 2 combined.

^c^Code in parentheses (eg, C04) indicates question number in instrument.

**Table 7:** Analysis of mean support for use of artificial intelligence (AI) in specific scenarios, 95% CIs, and *P* values for testing against a mean of 3. A score <3 represents support, and a score of >3 represents opposition^a^.

Domain and scenario			Estimated mean (95% CI)		*P* value^b^		Design effect
General—support for the development of AI (B01^c^)			2.35 (2.31-2.39)		<.001		1.83
**Health**							
	Data sharing for quality care (C04)	2.90 (2.83-2.98)		.01		1.65	
	Nonexplainable hospital algorithms (C03)	3.25 (3.18-3.32)		<.001		1.57	
	Deskilling physicians (C05)	3.39 (3.31-3.46)		<.001		1.62	
**Welfare**							
	Targeted compliance checking (D03)	2.98 (2.91-3.06)		.64		1.62	
	Automated parent support (contestability; D05)	3.06 (2.99-3.13)		.10		1.60	
	Nonexplainable job services (D04)	3.19 (3.12-3.26)		<.001		1.59	

^a^Means and CIs adjusted for weighting.

^b^*P* value for *t* test that the mean score was 3.0 using complex samples.

^c^Code in parentheses (eg, B01) indicates question number in instrument.

#### Statistical Significance of Differences Between Support in General and in Specific Scenarios

To further investigate these results, we statistically tested changes in responses between the general question (B01) and the more specific scenario questions (C03-C05 and D03-D05). Table 8 shows the percentage of those who changed between question B01 and each of the more specific scenarios and, of those who changed, what percentage changed to a more negative attitude. The change was tested against 50%, which corresponded to an equal change in a positive and negative direction.

Table 8 shows that the estimated percentage that answered differently between the general and the more specific questions was between 60.2% and 70.6%. Of those who changed, between 70.8% and 83% changed to a more negative response, and all of these changes were statistically significant. There was also a slight increase of 3% to 9% in neutral responses across specific scenarios compared with the general question.

**Table 8:** Estimated percentage of those who changed their response between the general question on the development of artificial intelligence and the specific scenarios and, of those who changed, the percentage that had a more negative attitude in the specific scenarios, with 95% CIs and the *P* value for the test of equal change in each direction^a^.

Domain and scenario			Percentage of those who changed		Percentage of those who changed becoming more negative (95% CI)		*P* value^b^		Design effect
**Health**									
	Data sharing for quality care (C04^c^)	60.2		70.8 (67.3-74)		<.001		1.59	
	Nonexplainable hospital algorithms (C03)	65.6		81.4 (78.6-83.9)		<.001		1.53	
	Deskilling physicians (C05)	70.6		83 (80.3-85.3)		<.001		1.56	
**Welfare**									
	Targeted compliance checking (D03)	63.8		71.9 (68.5-75)		<.001		1.65	
	Automated parent support (contestability; D05)	65		76.1 (73-78.9)		<.001		1.56	
	Nonexplainable job services (D04)	66.6		80.3 (77.5-82.9)		<.001		1.50	

^a^Percentages and CIs adjusted for weighting.

^b^Adjusted Pearson *F* test for equal proportions changing in each direction.

^c^Code in parentheses (eg, C04) indicates question number in instrument.

#### Statistical Significance of Differences in Support Between Scenarios

To assess the statistical significance of differences in support for different detailed scenarios, Table 9 shows estimates of the percentage of those who changed in response to pairs of questions and, of those who changed, the percentage expressing a more negative attitude on the second question and the associated test against 50%. Although most comparisons were within the health care or welfare domain, we asked about explainability in both the health care and welfare contexts, allowing us to make direct comparisons between this pair of questions.

As noted, the health care and welfare question blocks were randomized per participant, and the questions were randomized within blocks. As shown in Table 9, respondents did make different judgments in specific scenarios—there were statistically significant changes within all pairs except between the questions regarding explainability in health care and in welfare. Despite 45.7% of people changing their responses between these 2 questions, people changed their minds in both directions in approximately equal proportions. This suggests divided views on the importance of explainability in different scenarios. The differences between all health care scenarios were statistically significant. Answers on nonexplainability and deskilling were significantly different, and most were more negative than those on data sharing; answers on deskilling were significantly different, and most were more negative than those on nonexplainability. In addition, most changed their responses between these questions in the same direction. A similar pattern was seen in the welfare scenarios—a significant proportion of respondents changed their response among targeted compliance checking, automated parent support without contestability, and nonexplainable job services, in all cases to a more negative response. Again, most tended to change their responses among these questions in the same direction.

Comparisons of the general support and support in specific scenarios and between the scenarios were also analyzed using differences in the means, with similar conclusions.

**Table 9:** Estimated proportion of those who changed their response between 2 scenarios and, of those who changed, the percentage that expressed a more negative attitude in the second question, with 95% CIs and the *P* value for the test of equal change in each direction^a^.

Domain and scenarios compared		Percentage of those who changed	Percentage of those who changed becoming more negative (95% CI)	*P* value^b^	Design effect
**Health**					
	C03^c^ (explainability) vs C04^d^ (data sharing)	38.1	26.7 (22.7-31.1)	<.001	1.77
	C03 (explainability) vs C05^e^ (deskilling)	43.6	59.2 (55-63.3)	<.001	1.62
	C04 (data sharing) vs C05 (deskilling)	45.7	77.9 (74.2-81.2)	<.001	1.69
**Welfare**					
	D03^f^ (compliance checking) vs D04^g^ (explainability)	41.7	64.2 (60-68.2)	<.001	1.60
	D03 (compliance checking) vs D05^h^ (contestability)	45.1	55.6 (51.4-59.6)	.008	1.59
	D04 (explainability) vs D05 (contestability)	42.3	41.7 (37.6-45.9)	<.001	1.59
Explainability in health vs in welfare—C03 vs D04		45.7	46.1 (42-50.2)	.06	1.64

^a^Percentages and CIs adjusted for weighting.

^b^Adjusted Pearson *F* test for equal proportions changing in each direction.

^c^C03: nonexplainable hospital algorithms.

^d^C04: data sharing for quality care.

^e^C05: deskilling physicians.

^f^D03: targeted compliance checking.

^g^D04: nonexplainable job services.

^h^D05: automated parent support (contestability).

### Which Attributes of Health Care and Social Service AIs Were Most Important?

We provided 2 health care scenarios (C01 [machine diagnosis and treatment recommendations] and C02 [machine triage]) and 2 social service scenarios (D01 [automation of unemployment benefit decision-making] and D02 [chatbot advice about carer payments]). We asked respondents to rate the importance of different attributes of the AI system in each one, where the attributes reflected a key ethical, legal, or social dimension of the AI or its use. For health care scenarios, these attributes included responsibility for decision-making as this is central to medicolegal frameworks and professional autonomy. For welfare scenarios, they included personal tailoring as this is a key promise of automation and machine decision-making in welfare contexts.

**Figure 3:**
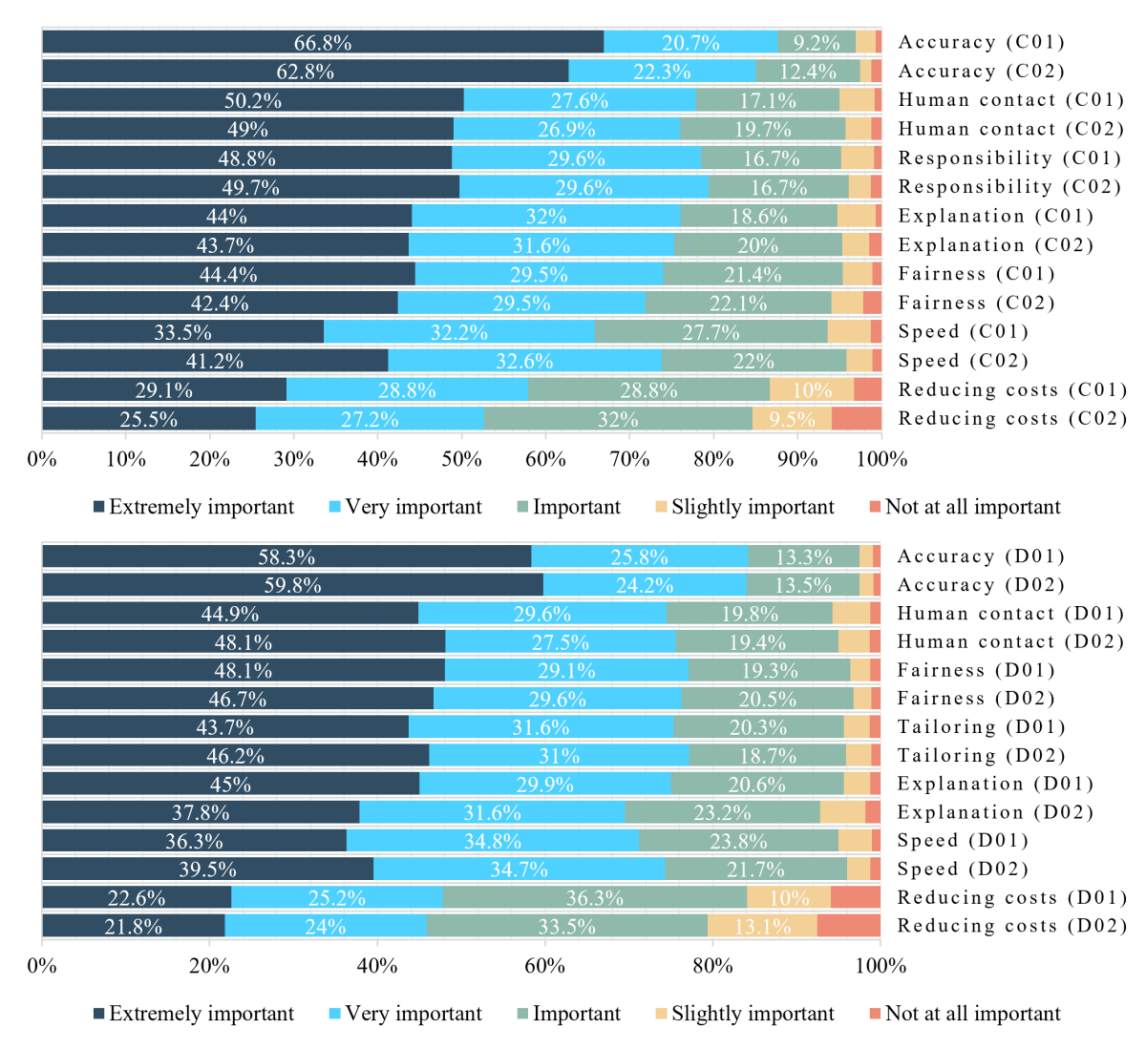
Responses to questions C01 to C02 versus D01 to D02: summary and comparison of health (C) and welfare (D) scenarios. Numerical estimates <10% are not given.

Figure 3 shows these responses to the health care and welfare scenarios to allow comparisons to be made between the distributions of the responses to any 2 questions assessing the same ethical or social dimension of AI. Multimedia Appendix 4 provides the detailed estimates of the proportions and the associated estimates of 95% CIs on estimated proportions for Figure 3.

Table 10 provides a summary of the importance that respondents ascribed to different attributes using mean scores, 95% CIs, and design effects. The response categories were scored from 1 for *extremely important* to 5 for *not at all important*; thus, lower scores indicate more importance. All means were <3, the midpoint of the scale; t tests against a mean of 3 were statistically significant with *P*<.001, indicating that more of the distribution of responses was in the extremely or very important categories. The attributes in Table 10 are in ascending order of means, that is, from most to least important (where the most important value is presented first).

As shown in Figure 3 and Table 10, there were distinctions between attributes. In all 4 scenarios, accuracy was rated as most important on average (1.49-1.61), and the ability of an AI system to reduce system costs was rated as least important (2.30-2.60), especially in health care. After accuracy, fairness was the second most important attribute in both social service scenarios (1.80 and 1.81) but, in the health care scenarios, it placed lower relative to other attributes (1.87 and 1.94). After accuracy, responsibility and human contact were the next most important in both health care scenarios. Speed was slightly more important in a health care triage scenario (1.90) than in a medical testing scenario (2.08).

Table 11 compares the mean responses to the attribute questions for the 2 health care scenarios (C01 vs C02) and the 2 welfare scenarios (D01 vs D02) to assess whether there were differences in importance in specific scenarios. In these comparisons, a negative estimate of the difference implies more importance for the first listed question, and a positive difference implies more importance for the second listed question. Table 12 provides further analysis, including statistical significance testing, of shifts in responses to the questions. Taken together, these tables show that, among the health care scenarios, the only statistically significant differences were in relation to speed (more important in triage) and reducing costs (more important in decision support). In the social service scenarios, more statistically significant differences were found, with explanation and cost reduction being more important in automating unemployment benefits and human contact, speed, and personal tailoring being more important in receiving automated carer support advice.

**Table 10:** Means, 95% CIs, and design effects for importance of values.

		Estimate of the mean^a^ (95% CI)	Design effect	
**C01^b^ —machine reads medical test, diagnoses, and recommends treatment**				
	Accuracy	1.49 (1.46-1.53)	1.98	
	Human contact	1.78 (1.74-1.81)	1.95	
	Responsibility	1.78 (1.75-1.82)	1.98	
	Explanation	1.86 (1.82-1.90)	1.96	
	Fairness	1.87 (1.83-1.91)	1.91	
	Speed	2.08 (2.04-2.12)	1.88	
	Reducing costs	2.30 (2.25-2.34)	1.92	
**C02—machine triages when you are unwell**				
	Accuracy	1.56 (1.51-1.61)	1.73	
	Responsibility	1.76 (1.71-1.81)	1.75	
	Human contact	1.81 (1.75-1.86)	1.72	
	Explanation	1.87 (1.82-1.93)	1.76	
	Speed	1.90 (1.85-1.95)	1.64	
	Fairness	1.94 (1.88-2.00)	1.81	
	Reducing costs	2.43 (2.36-2.50)	1.74	
**D01—machine processes application for unemployment benefits (data sharing required)**				
	Accuracy	1.61 (1.56-1.65)	1.53	
	Fairness	1.80 (1.75-1.85)	1.56	
	Explanation	1.86 (1.80-1.91)	1.61	
	Personal tailoring	1.87 (1.82-1.92)	1.58	
	Human contact	1.88 (1.82-1.93)	1.54	
	Speed	1.99 (1.93-2.04)	1.58	
	Reducing costs	2.51 (2.45-2.58)	1.59	
**D02—chatbot advises about carer payments**				
	Accuracy	1.60 (1.55-1.64)	1.6	
	Fairness	1.81 (1.76-1.87)	1.68	
	Personal tailoring	1.82 (1.77-1.87)	1.67	
	Human contact	1.83 (1.77-1.88)	1.63	
	Speed	1.91 (1.86-1.97)	1.71	
	Explanation	2.02 (1.96-2.08)	1.72	
	Reducing costs	2.60 (2.54-2.67)	1.71	

^a^Means and CIs adjusted for weighting.

^b^Code (eg, C01) indicates question number in instrument.

**Table 11:** Differences in mean responses on importance of attributes between 2 scenarios^a^.

Domain and attribute			Mean difference (95% CI)		*P* value^b^		Design effect
**Health—C01^c^ vs C02^d^**							
	Explanation	−0.001 (−0.048 to 0.046)		.96		1.89	
	Speed	0.082 (0.040 to 0.123)		<.001		1.51	
	Accuracy	−0.009 (−0.052 to 0.033)		.67		1.91	
	Human contact	−0.012 (−0.060 to 0.036)		.63		2.12	
	Responsibility	0.007 (−0.035 to 0.050)		.73		1.88	
	Reducing costs	−0.111 (−0.162 to −0.060)		<.001		1.99	
	Fairness	−0.035 (−0.081 to 0.011)		.13		1.93	
**Welfare—D01^e^ vs D02^f^**							
	Explanation	−0.164 (−0.215 to −0.113)		<.001		1.64	
	Speed	0.070 (0.029 to 0.111)		<.001		1.59	
	Accuracy	0.012 (−0.023 to 0.048)		.50		1.42	
	Human contact	0.049 (0.009 to 0.089)		.02		1.48	
	Personal tailoring	0.048 (0.006 to 0.090)		.02		1.58	
	Reducing costs	−0.091 (−0.136 to −0.046)		<.001		1.54	
	Fairness	−0.018 (−0.059 to 0.029)		.38		1.72	

^a^Means and CIs adjusted for weighting.

^b^*P* value for *t* test that the mean difference was 0 using complex samples.

^c^C01: machine reads medical test, diagnoses, and recommends treatment.

^d^C02: machine triages when you are unwell.

^e^D01: machine processes application for unemployment benefits (data sharing required).

^f^D02: chatbot advises about carer payments.

**Table 12:** Estimated percentages of those who changed their responses on importance of values between 2 scenarios and, of those, the percentage that ranked the value to be more important in the first question than in the second question (C01 vs C02 or D01 vs D02), with associated 95% CIs and the *P* value for the test of equal cell proportions^a^.

Domain and values			Percentage of those who changed		Percentage ranking the value as more important in C01 (vs C02) or D01 (vs D02) (95% CI)		*P* value^b^		Design effect
**Health—C01^c^ vs C02^d^**									
	Explanation	34.3		47.6 (42.8-52.4)		.33		1.68	
	Speed	34.9		39.5 (35.2-44.1)		<.001		1.52	
	Accuracy	25.1		49.5 (43.8-55.2)		.86		1.68	
	Human contact	29.9		50.3 (45-55.5)		.92		1.70	
	Responsibility	28.3		47.7 (42.5-53)		.40		1.69	
	Reducing costs	33		59.2 (54.3-63.9)		<.001		1.66	
	Fairness	29.3		53.7 (48.5-58.8)		.16		1.66	
**Welfare—D01^e^ vs D02^f^**									
	Explanation	39.6		63.7 (59.4-67.7)		<.001		1.55	
	Speed	32.7		41.8 (37-46.6)		.001		1.66	
	Accuracy	26.4		48.4 (43.2-53.7)		.56		1.57	
	Human contact	30.7		43.9 (39.1-48.8)		.02		1.64	
	Personal tailoring	33.1		43.9 (39.1-48.8)		.01		1.69	
	Reducing costs	35.1		58.8 (54.3-63.1)		<.001		1.58	
	Fairness	27.1		51.7 (46.3-57.1)		.53		1.70	

^a^Percentages and CIs adjusted for weighting.

^b^Adjusted Pearson *F* test for equal proportions.

^c^C01: machine reads medical test, diagnoses, and recommends treatment.

^d^C02: machine triages when you are unwell.

^e^D01: machine processes application for unemployment benefits (data sharing required).

^f^D02: chatbot advises about carer payments.

### Final Bundled Attribute Trade-off of AI and Human Attributes

**Figure 4:**
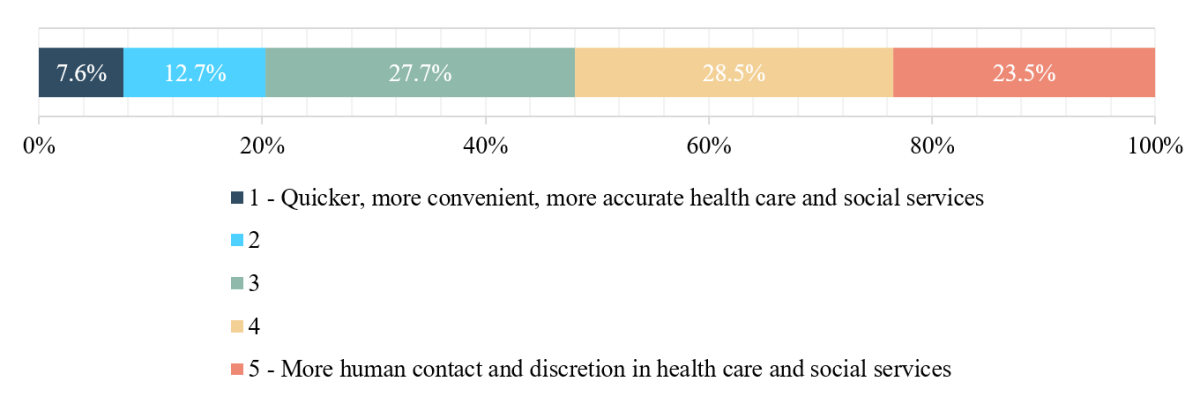
Responses to question E01: speed, accuracy, and convenience versus human contact and discretion.

Figure 4 shows the estimated percentages for the final bundled trade-off question (E01), where respondents were asked to weigh speed, convenience, and accuracy against human contact and discretion. Table 13 provides the estimated percentages, mean scores, and 95% CIs. These results show that human attributes were generally valued more, as indicated by a mean score >3. The estimated proportion of those who preferred the machine attributes (categories 1 or 2) was 20.3%, whereas, for human attributes (categories 4 or 5), it was 52%; 27.7% selected a middle position.

**Table 13:** Speed, accuracy, and convenience versus human contact and discretion; estimated percentages; and 95% CIs for responses to question E01^a^.

	Estimate (95% CI)
1: speed, convenience, and accuracy	7.6 (6.2-9.1)
2	12.7 (11-14.7)
3	27.7 (25.3-30.3)
4	28.5 (26.1-31.1)
5: human contact and discretion	23.5 (21.2-26)
Mean score^b^	3.38 (3.41-3.54)

^a^Percentages and CIs adjusted for weighting.

^b^*P*<.001 for testing that the mean score was 3; design effect=1.602.

## Discussion

### Principal Findings

#### Overview

The AVA-AI study has created one of the first large, robust data sets reflecting public views on the potential use of AI in health care and social services, with particular attention to the ELSI of those technologies. Future studies will provide a greater breakdown of the variation in responses among different population subgroups. This analysis focused on answering 3 key questions: how judgments in general compare with judgments in particular, how judgments about use in health care compare with judgments about use in social services, and whether judgments differ when ELSI differ.

#### General Versus Particular Judgments About AI

Our first general question about support for or opposition to AI was taken from the 2018 survey of the American public by Zhang and Dafoe [26], which included 2000 respondents and used a similar weighting methodology; the Monash Data Futures survey [27] also included this question and surveyed 2019 respondents. Owing to our methodology, we asked this question to 4448 respondents. Table 14 compares these results—as the Monash survey reports combined *all support* and *all oppose* categories only, we have done the same. Both the AVA-AI study and the Monash survey suggest more positive general views in Australia than in the United States, although the results of the AVA-AI study are less positive than those of the Monash survey. Speculative reasons for this difference could include more prominent public discourse regarding harms from AI deployment in the US context (eg, in policing, justice, warfare, and the retail sector) or, more tentatively, that, in the 2 years between the surveys (mid-2018 for the study by Zhang and Dafoe [26] vs March 2020-April 2020 for both the AVA-AI study and the Monash survey), Australians may have had additional positive experiences of the everyday AI described in that question (eg, language translation, spam filters, and streaming content suggestions).

As a minority of AVA-AI study respondents began the survey with negative general views on AI and >60% expressed support, any negative judgments expressed seem likely to be a response to the details of the scenarios presented rather than reflect prejudice against or fear of AI in general. When asked about specific scenarios for AI use, respondents were consistently more negative—the reduction in support between the general question and all 6 specific scenarios was statistically significant, and support expressed in the specific scenarios dropped between 17 and 33 percentage points. The simple opening *support-or-oppose* question presented familiar, helpful everyday examples of AI in use and did not demonstrate any downsides of AI. In contrast, the detailed scenario questions were designed for balance. Each question emphasized that AI could both improve services (eg, make them quicker, more convenient, and more accurate) and have downsides (eg, reduced explainability, contestability, and privacy; unfair burdens on minorities; or human deskilling). On the basis of our findings, we hypothesize that members of the general public may remain broadly unaware of the potential downsides of AI in use and that some of these downsides (eg, deskilling) matter more to them than others (eg, privacy). We did not test the level of awareness of ELSI problems with AI—this is a potential direction for future research. Participants’ more negative judgments in the case-specific questions also empirically reinforce what has already been argued in the literature: that the ELSI of AI applications need to be considered in the context of detailed cases.

**Table 14:** Comparison of findings from the studies by Zhang and Dafoe [26] and the Monash Data Futures Institute [27] and from the Australian Values and Attitudes on Artificial Intelligence (AVA-AI): How much do you support or oppose the development of artificial intelligence?

	Zhang and Dafoe [26] (2018), weighted %	Monash Data Futures Institute [27] (2020), weighted % by age only	AVA-AI (2020), weighted %
Strongly or somewhat support	40.94	62.4	60.3
Neither support nor oppose	27.84	23	21.9
Strongly or somewhat oppose	21.69	10.5	13.4
I don’t know	9.54	4.1	4.4

#### Judgments About Health Care Versus Judgments About Social Services

Respondents had slightly stronger, more diverse, and more negative views on using AI in health care as opposed to in social services. This may be because they themselves have more direct experience of using health care or consider health care more relevant to them; alternatively, respondents may consider health care to be a higher-stakes service for which they are less tolerant of social or ethical wrongs or harms. Again, respondents in the AVA-AI study were less strongly supportive than respondents in the Monash survey, expressing 27% to 43% support for health care scenarios and 31% to 39% support for social service scenarios. In the Monash survey, respondents were asked to rate their support or opposition to *the application of artificial intelligence to social, humanitarian and environmental challenges*. The areas that received the most support—>75% of respondents—were *health* and *medicine*, whereas the areas that received the least support (although still >60%) included *equality and inclusion* and *public and social sector management*.

The differing responses to the 2 surveys may arise from the framing of the questions. The Monash questions were framed optimistically and presented no downsides; the AVA-AI questions presented both benefits and downsides or burdens. In health care, we held effectiveness and health benefits against requirements to share data, nonexplainability, and clinical deskilling. In social services, we held the accuracy and consistency of predictions and decisions against the potential for overtargeting, poor contestability, and nonexplainability. The differences in responses between the 2 surveys may show that the ethical and social risks of AI matter to people and will make a difference in their evaluations.

#### Do Judgments Differ When ELSI Differ?

The respondents clearly made judgments about the ELSI of AI. Although all ELSI were considered important, this was by degree. Respondents made quite finely graded judgments that intuitively aligned with the characteristics of the scenarios, suggesting both that they took the questions seriously and that different attributes will be differently important in different cases. For example, speed was more important in triage, where time is critical, than in diagnosis. Explanation was more important in automating unemployment benefits than in an information chatbot, which would be consistent with the view that people deserve to know why they do or do not receive payments. Human contact, personal tailoring, and speed were more important for the chatbot than for the benefits system, possibly reflecting that chatbot interactions are short and information-heavy and that people want a human to talk to if the automated system fails.

Two things were consistent: accuracy was always the most highly valued, and reducing costs was always the least highly valued across health care and social services. The lack of any significant difference in the importance of accuracy across scenarios suggests that this is an entry-level requirement for the use of AI (although defining *accuracy* in different contexts is not straightforward). The lower importance given to cost reduction may reflect a general rejection of instrumental decision-making in policy and of cost-based arguments in public services. Contextual factors include Australia’s publicly funded health care system being *popular and entrenched* [53] and that, despite holding negative views on welfare recipients, the Australian public remains similarly supportive of the welfare system as a whole [54].

Fairness was more important in social services than in health care. This may reflect the centrality of the concept of procedural fairness—that is, the fairness of the decision-making process—in social service administration, particularly within Australia’s bureaucratic and rule-bound welfare system [55]. It may also reflect heightened concern for issues of fairness in light of the public controversy surrounding the robodebt program, which centered on the legality, accuracy, and fairness of the program’s debt calculations [23]. Perhaps the most deliverable promise of AI is increased speed, but this was not highly valued by respondents in any of the scenarios presented.

Knowing who is responsible for decisions, especially any mistakes made, was consistently important in health care, suggesting that the regulatory and ethical governance challenges in health care AI will matter to the public. Human contact was also important in health care. Prominent health care AI advocates have suggested that the core benefit of health care AI is its ability to release clinicians from mundane duties, freeing them to engage more deeply in care work [56]. However, the digitization of health care in some contexts has had the opposite effect, overburdening clinicians with data management and system requirements that alienate them from patient care [57]. This will be a key challenge to manage if health care AI is to deliver on its promises. Relatedly, respondents rejected medical deskilling most strongly among our 3 health care scenarios. This resonates with empirical research suggesting that people strongly value the preservation of human oversight for AI decision-making but also suggests the need for more work on what kinds of deskilling matter most as deskilling is highly likely to occur as automation increases. As in other research, participants were weakly supportive of sharing their health data with a learning health system if it delivered better quality care [58], although qualitative and deliberative research suggests that this support is likely to be conditional [59]. Respondents were also weakly supportive of algorithmic targeting of welfare compliance checking to high-risk groups if this saved money and reduced the number of checks on other people, which may reflect an on-balance judgment about proportionality or may simply reflect the aforementioned negative views on welfare recipients.

We asked about explainability in both health care and welfare scenarios and contestability in welfare scenarios. Respondents expressed an on-balance opposition to both health care and welfare AIs that were not explainable to relevant professionals. However, different respondents valued explainability differently in health care and welfare scenarios, suggesting that there may be some divergence in people’s views on the domains in which explanation is more important. There was also an on-balance opposition to noncontestability in welfare scenarios, which reinforces support for processes of review and appeal when welfare decision-making is automated.

When asked to make an on-balance judgment about the *bundle* of attributes most commonly associated with machines versus with humans, respondents strongly preferred human attributes. Although they considered attributes such as accuracy to be important if an AI system was to be implemented, they still highly valued human support and connection and were not prepared to give them up in exchange for accuracy (despite the accuracy of AI being highly valued in itself). This suggests the importance of pursuing an augmentation rather than a replacement role for AI in both health care and social services. For all of these findings, further qualitative research is needed to better understand the reasons underpinning people’s judgments.

### Limitations

To the best of the authors’ knowledge, this study is one of the largest and most robust surveys of public attitudes toward health care and welfare AI to date. The methodological approach taken allowed for the collection of detailed information on attitudes for a substantial sample using a relatively low-cost web-based panel while compensating for the potential biases in the creation of such a panel. Although the results suggest that respondents were able to engage with the details of the questions, the relatively low level of knowledge of AI in the community and the speculative nature of the questions mean that people’s responses to a direct experience of AI may differ from their responses in this survey. A strength of our design was the use of questions that were deliberately structured to present both the potential benefits and the potential burdens or harms of AI while attempting to maintain neutral sentiment and avoid normative valence in the language used. The survey was conducted before the onset of the COVID-19 pandemic, which initiated the rapid digitization of many health care and social services; it is possible that responses would be different if the survey were repeated today.

### Conclusions

Australians support the idea of AI in a general sense, but their support diminishes when considering the details of particular scenarios and the potential harms or burdens that may accompany any promised benefits. Respondents consistently rated the accuracy of performance as the most important attribute in an AI system, but only 1 in 5 valued the speed, accuracy, and convenience of AI systems more than continued human contact and discretion in service provision. Overall, this study suggests that the ethical and social dimensions of AI systems matter to Australians and that Australians want AI systems to augment rather than replace humans in the provision of both health care and social services and to reflect human values. Meaningful engagement and participation of ethicists, social scientists, and the public can highlight what harms and wrongs are most important to avoid in all stages of the development and implementation of AI, including in sensitive and value-laden domains such as health care and social services.

## Appendix

Multimedia Appendix 1 Survey.

Multimedia Appendix 2 Extended sample composition.

Multimedia Appendix 3 Support or opposition in specific artificial intelligence scenarios.

Multimedia Appendix 4 Importance of health (C) and welfare (D) scenarios.

## Abbreviations

AI: artificial intelligence
AVA-AI: Australian Values and Attitudes on Artificial Intelligence
ELSI: ethical, legal, and social implications
LIA: Life in Australia
