# Supplementary figures and images for: The Adoption of Artificial Intelligence in Health Care and Social Services in Australia: Findings From a Methodologically Innovative National Survey of Values and Attitudes (the AVA-AI Study)
Source: J Med Internet Res. 2022 Aug 22;24(8):e37611. doi: 10.2196/37611 (PMC9446139; doi:10.2196/37611)

**
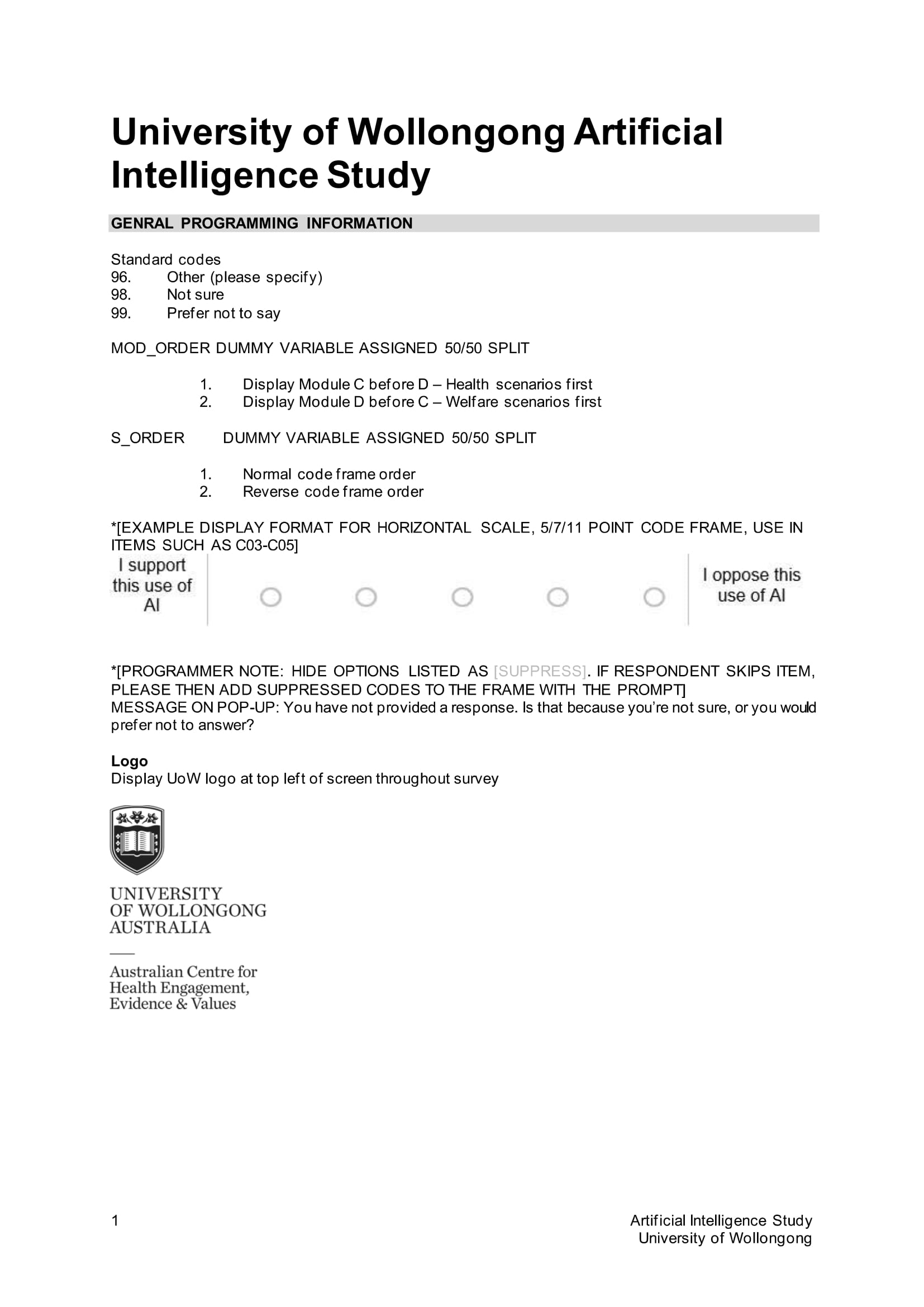

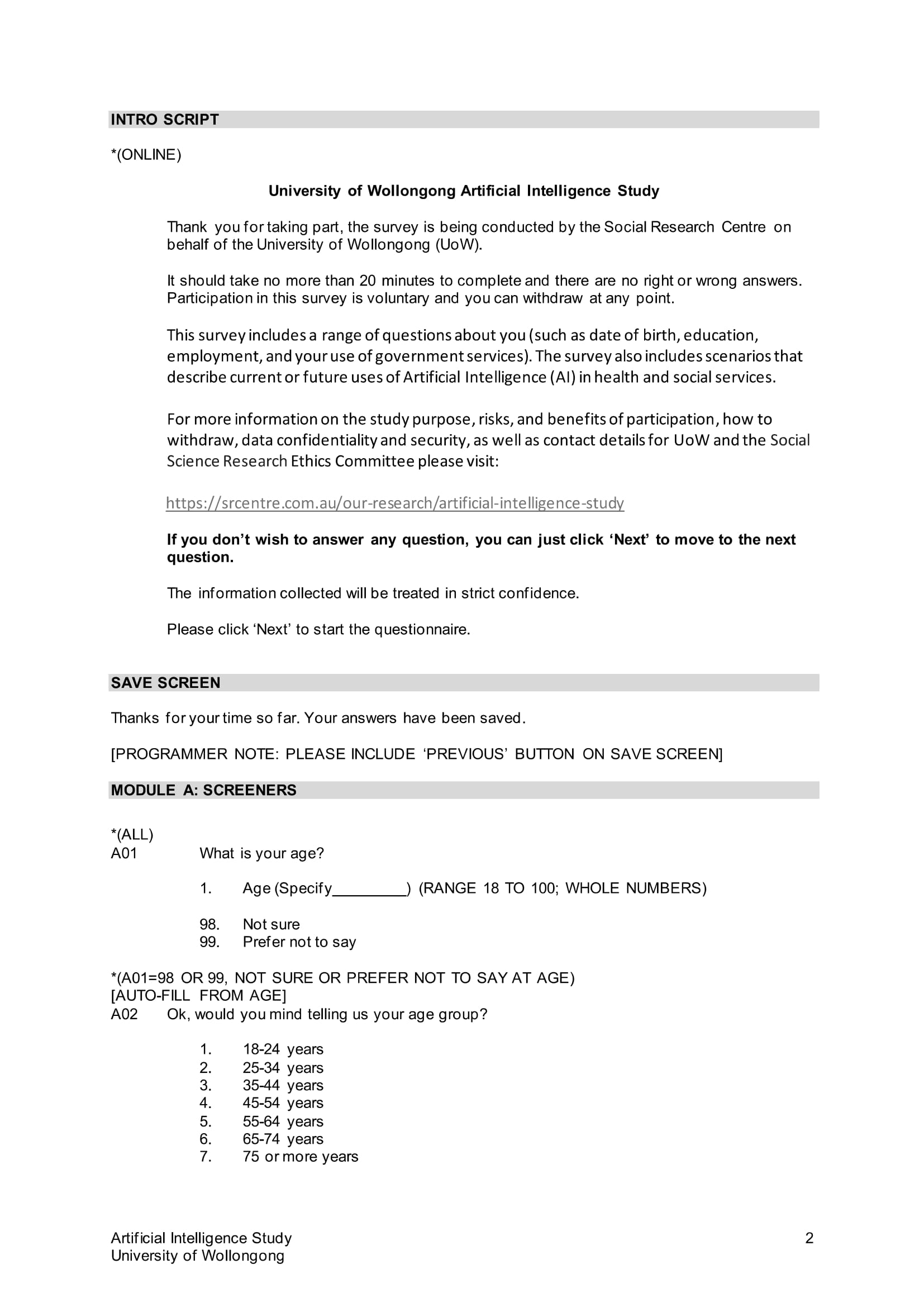

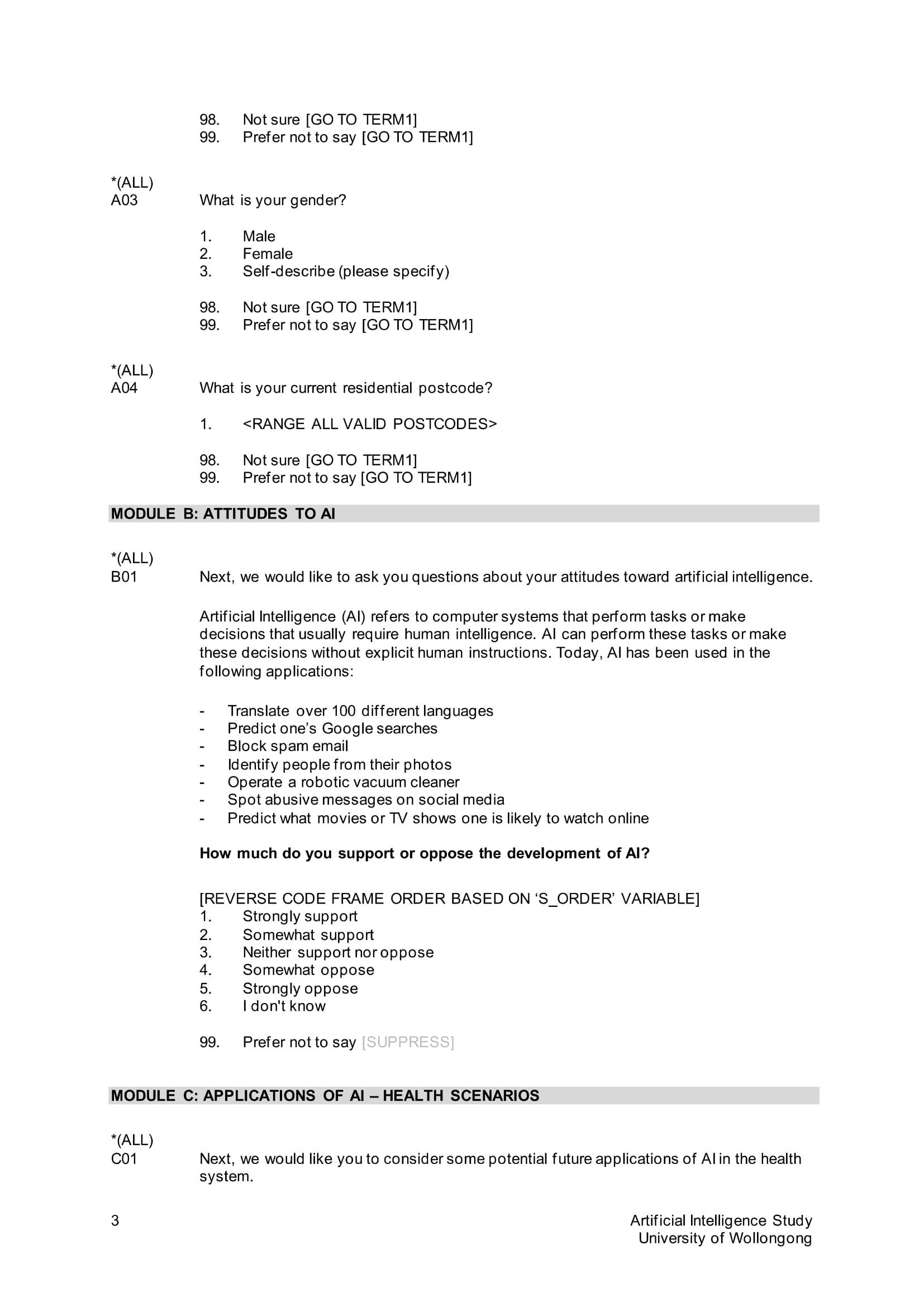

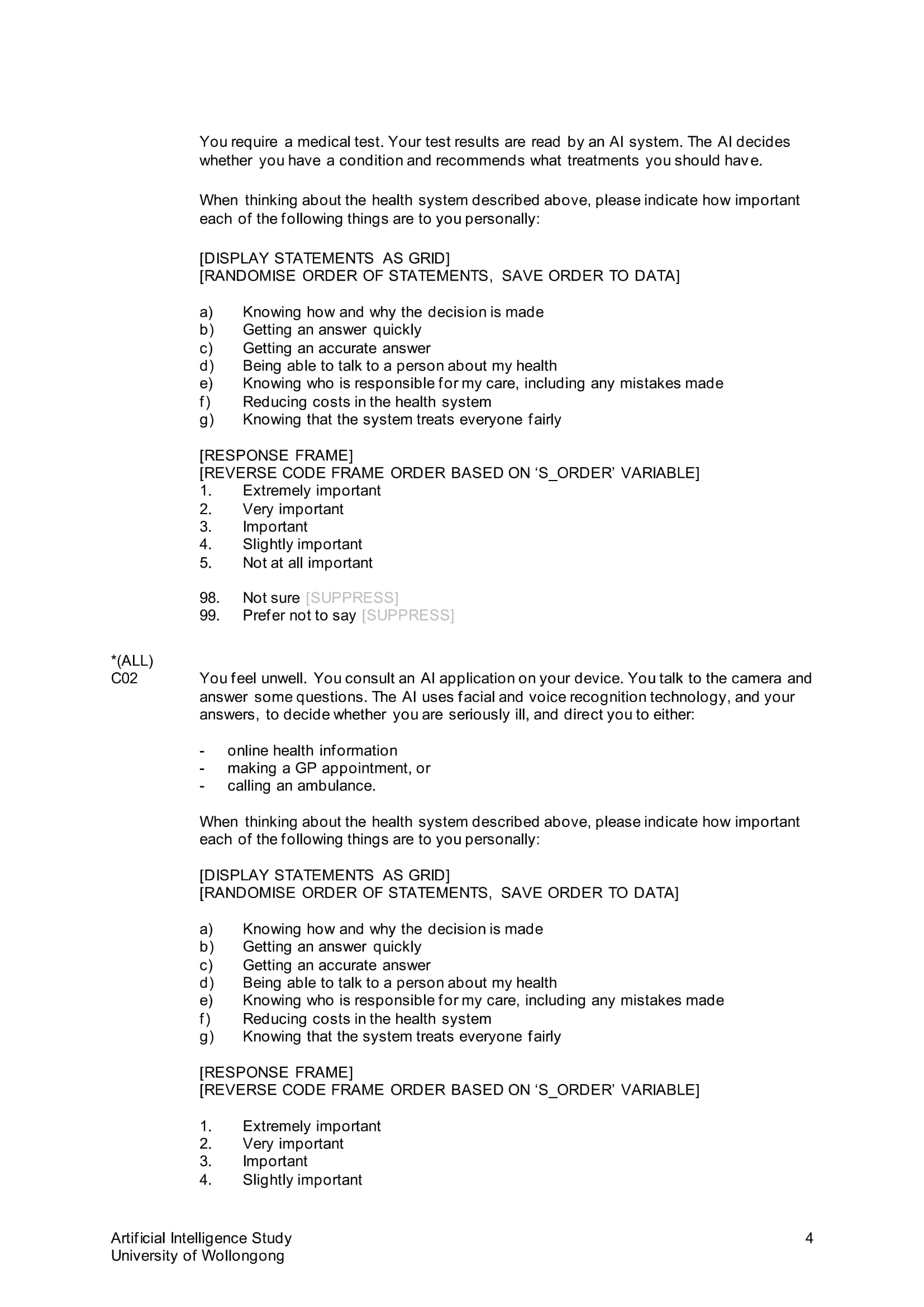

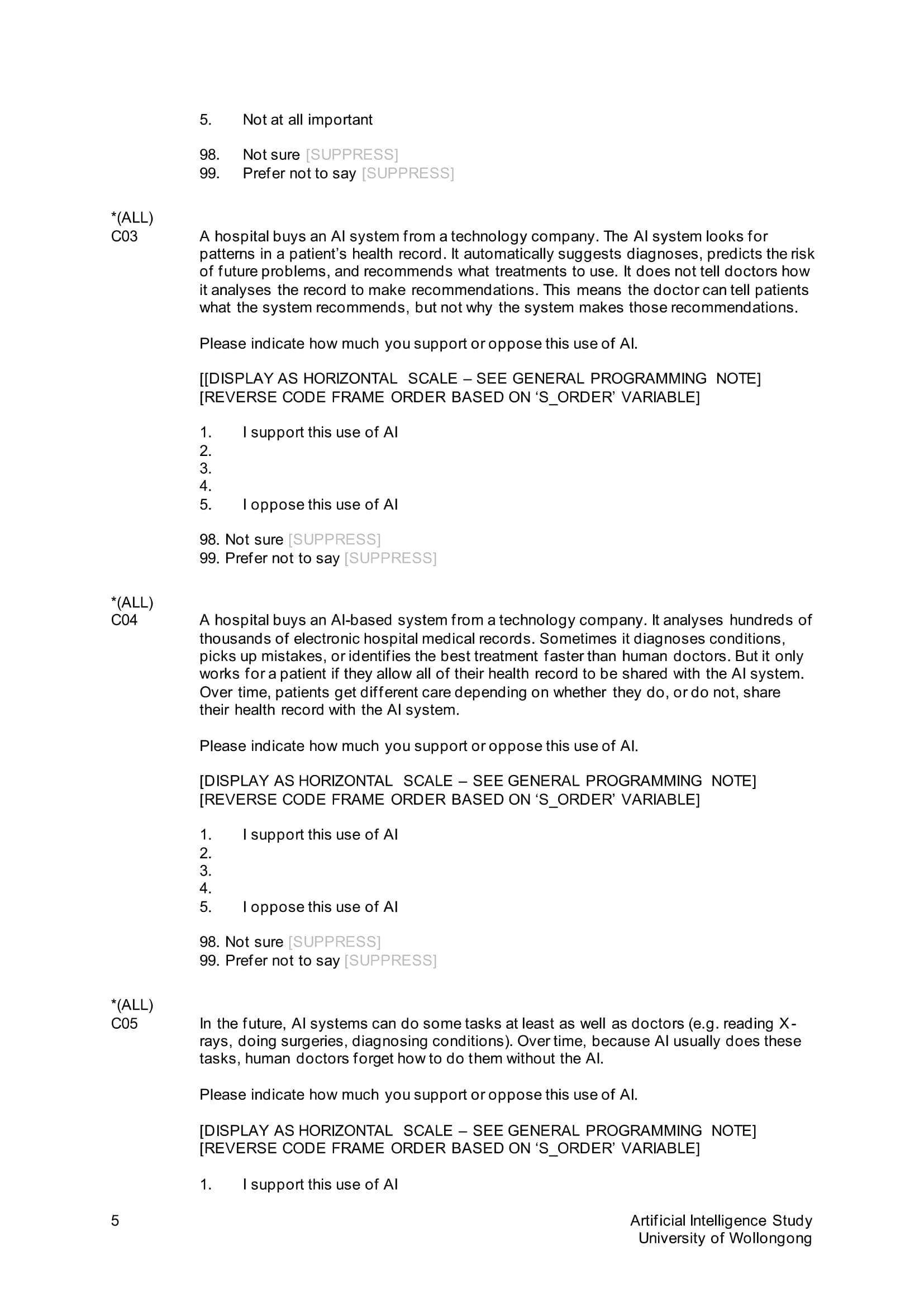

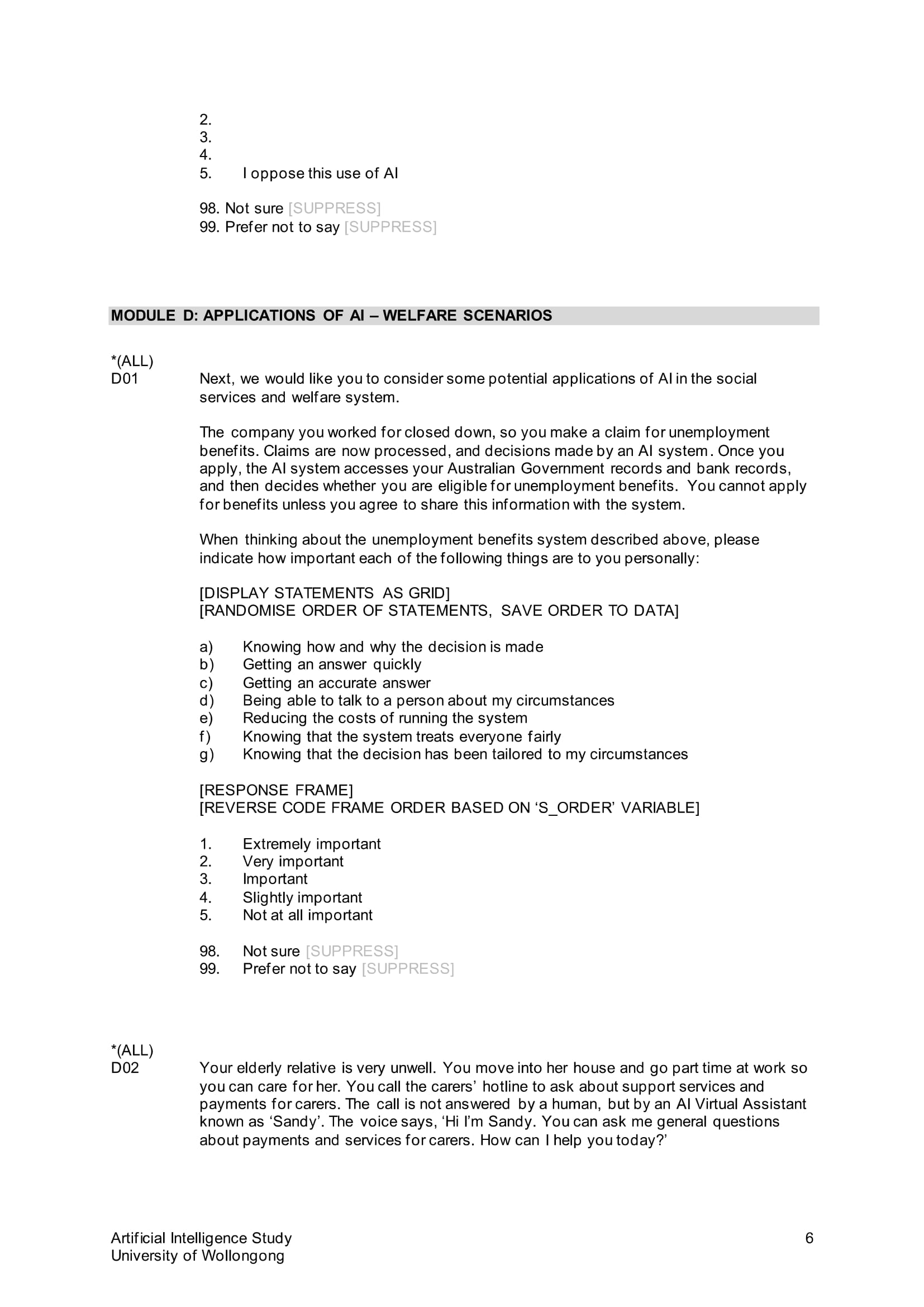

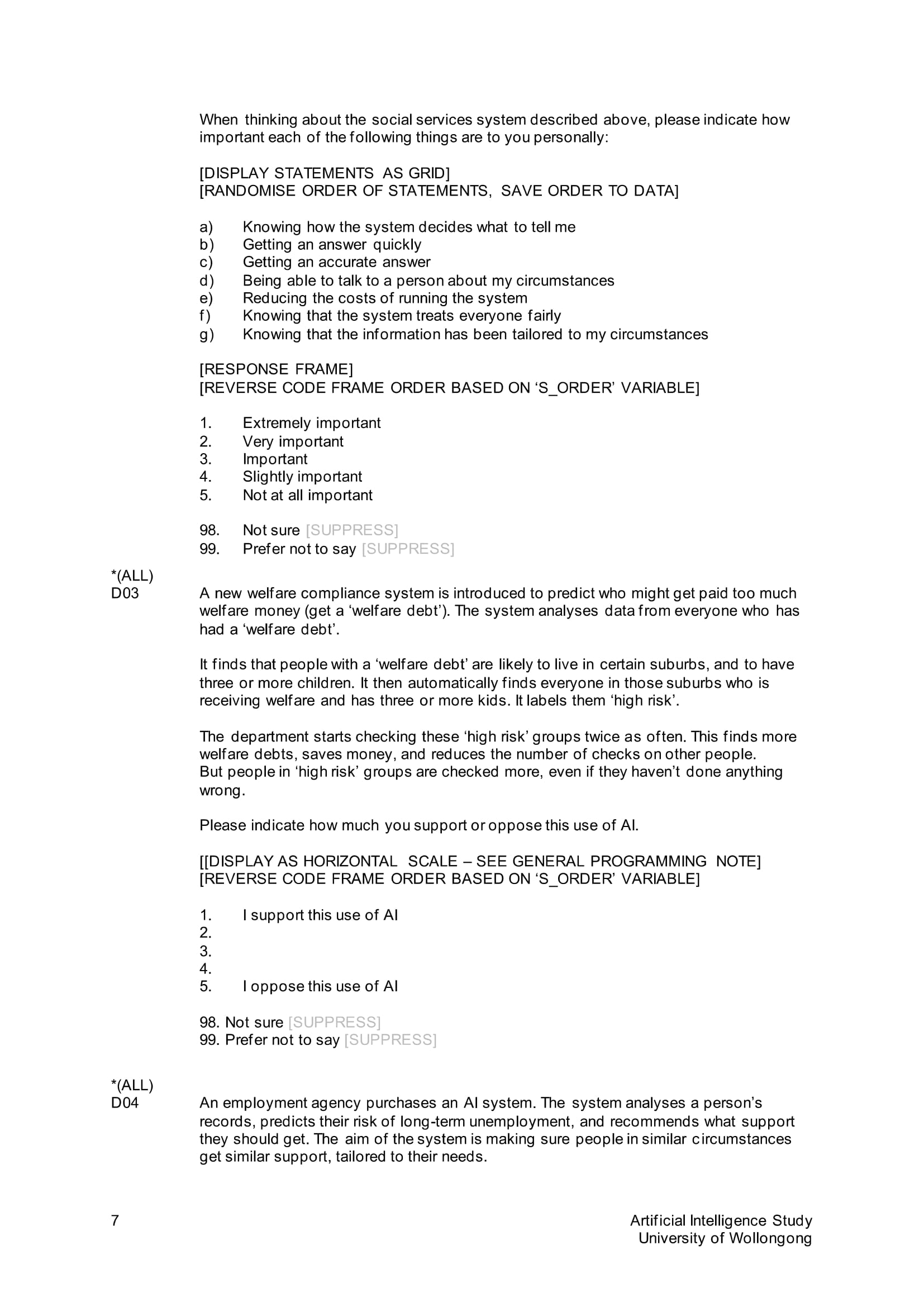

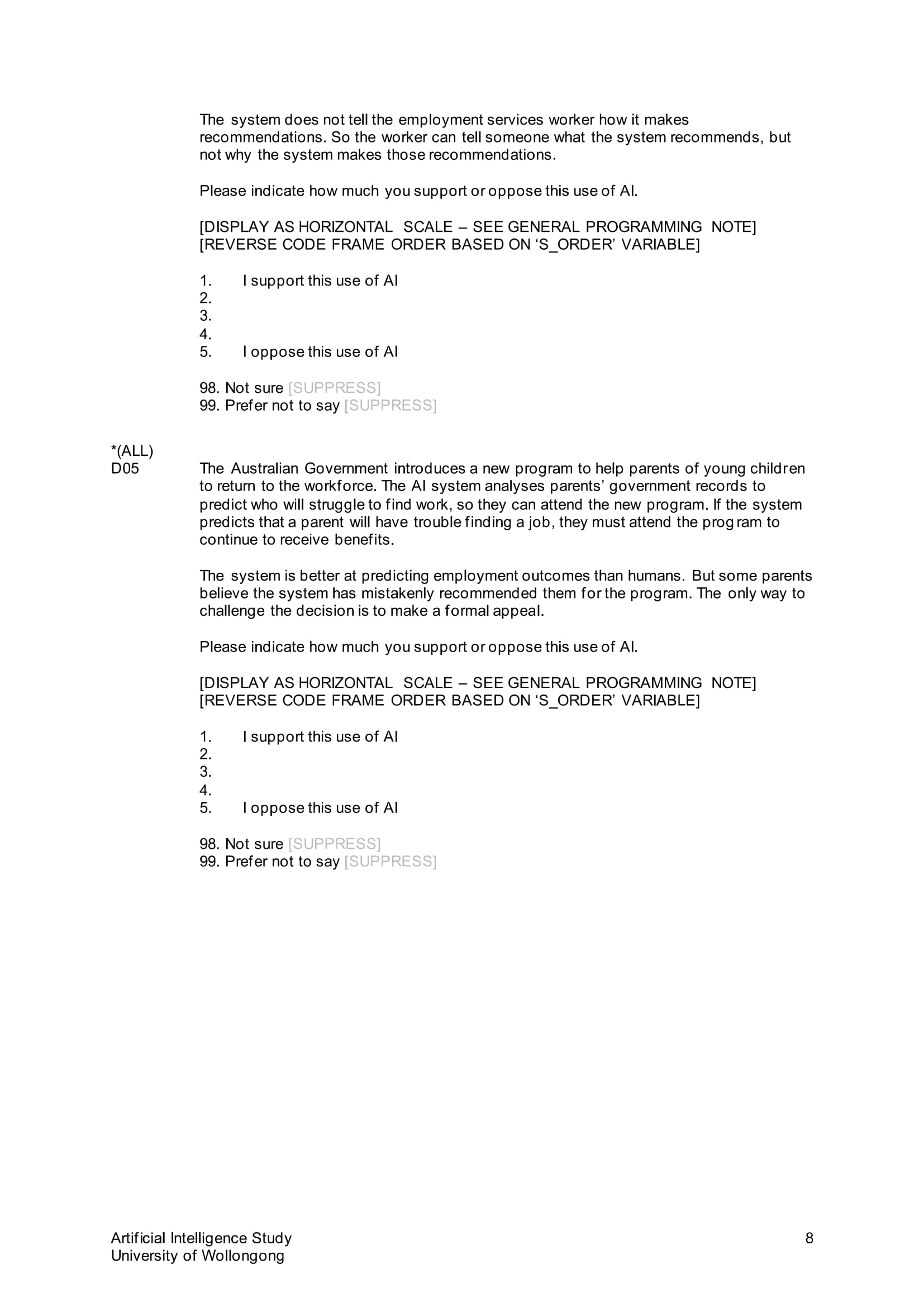

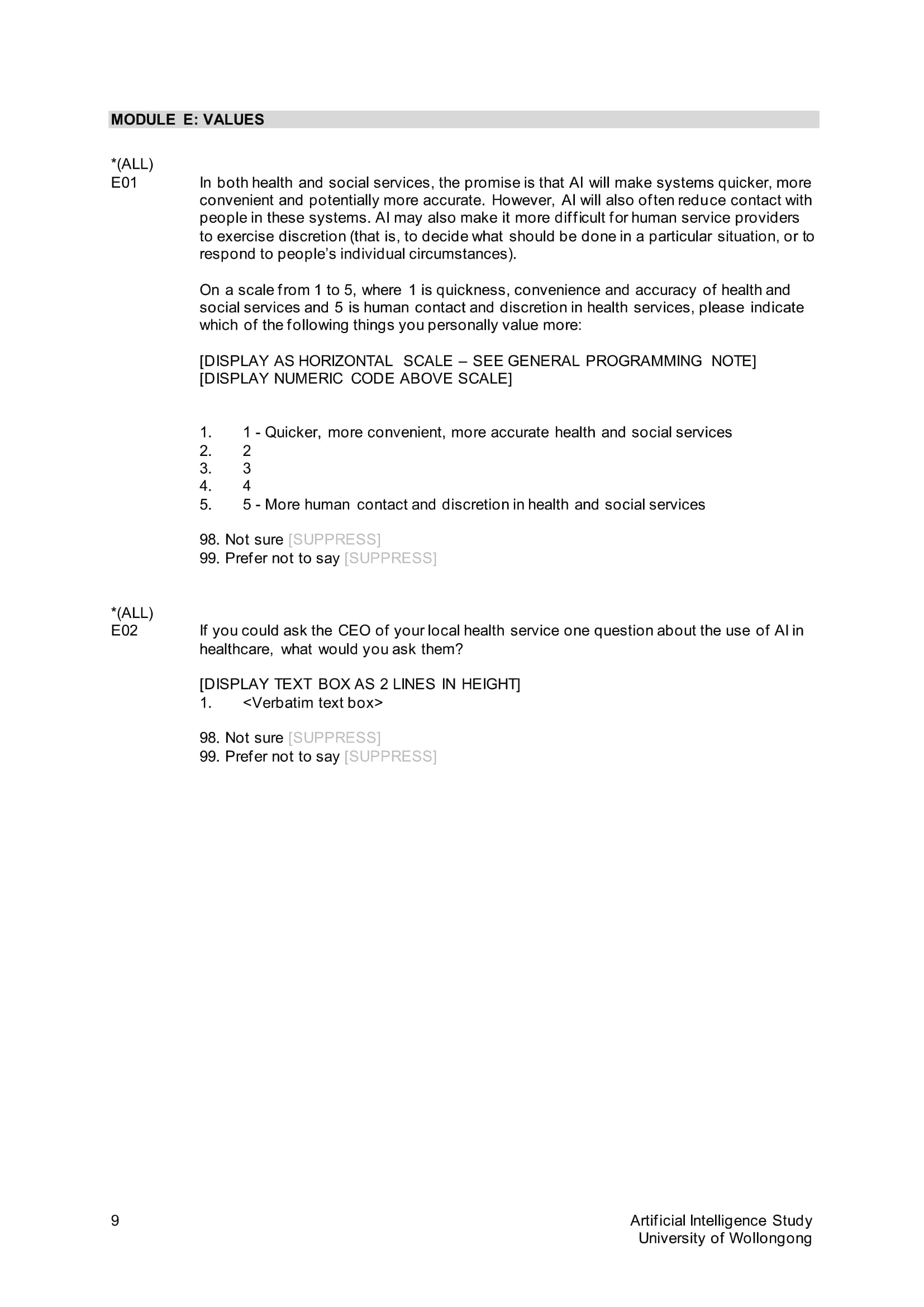

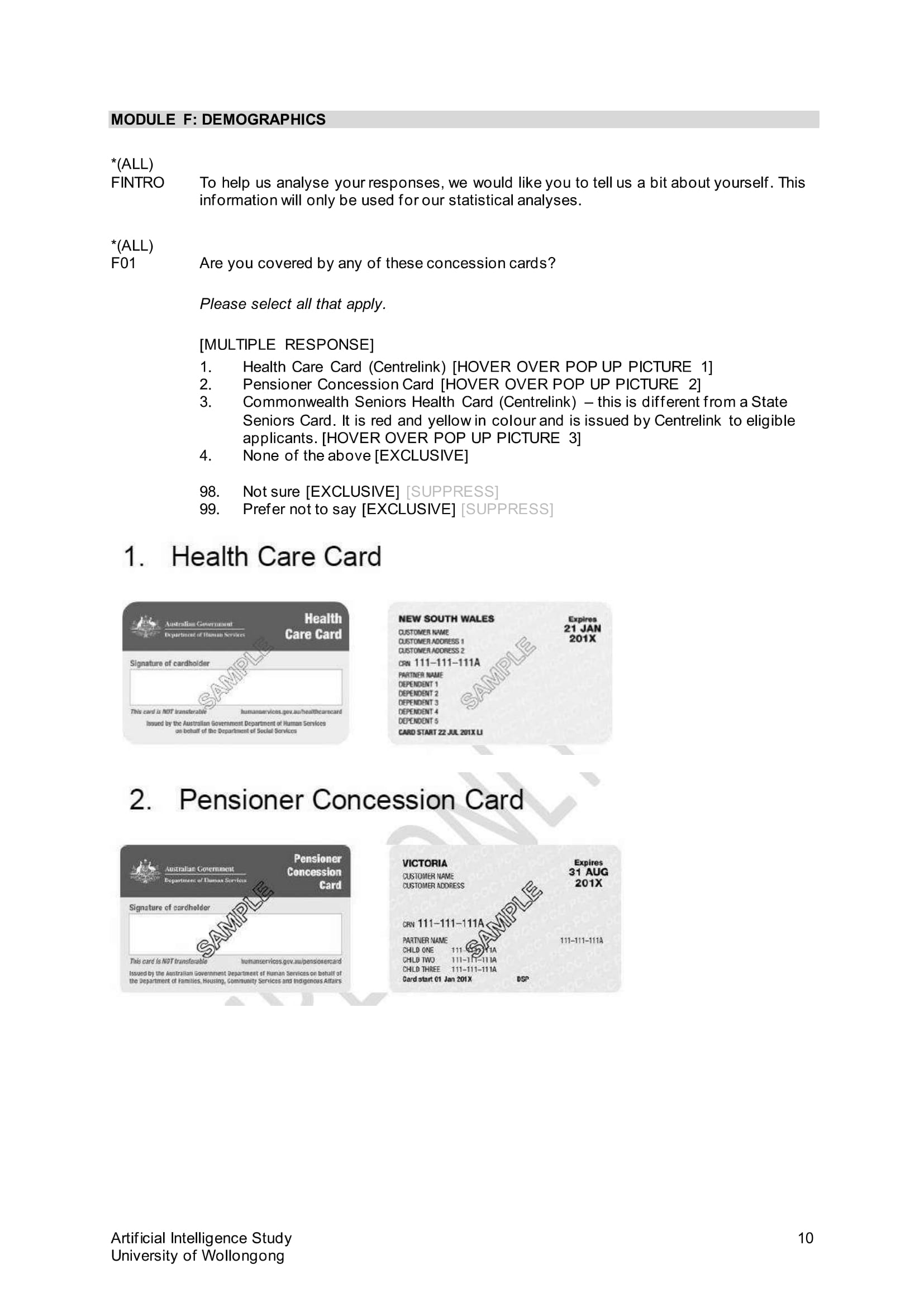

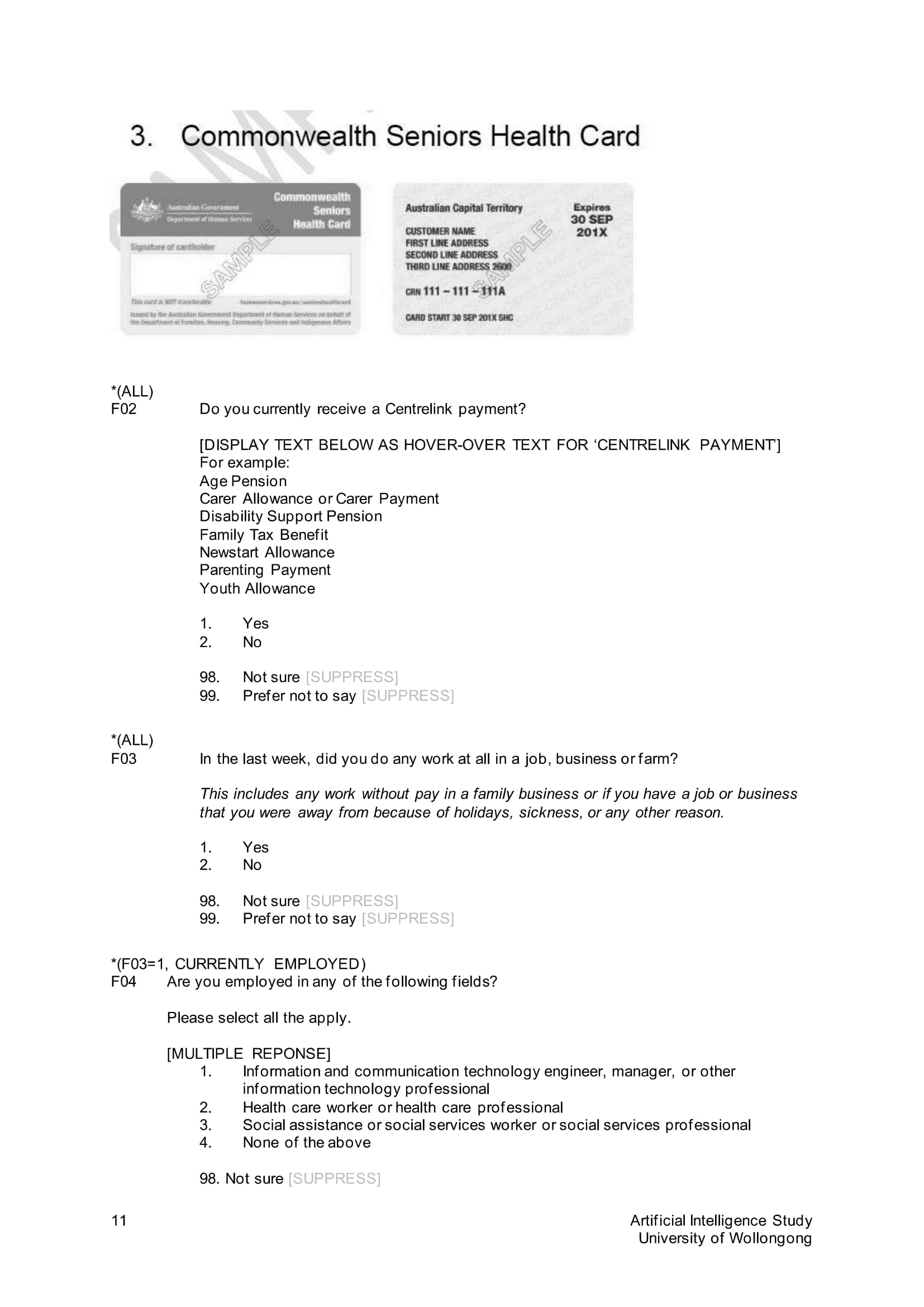

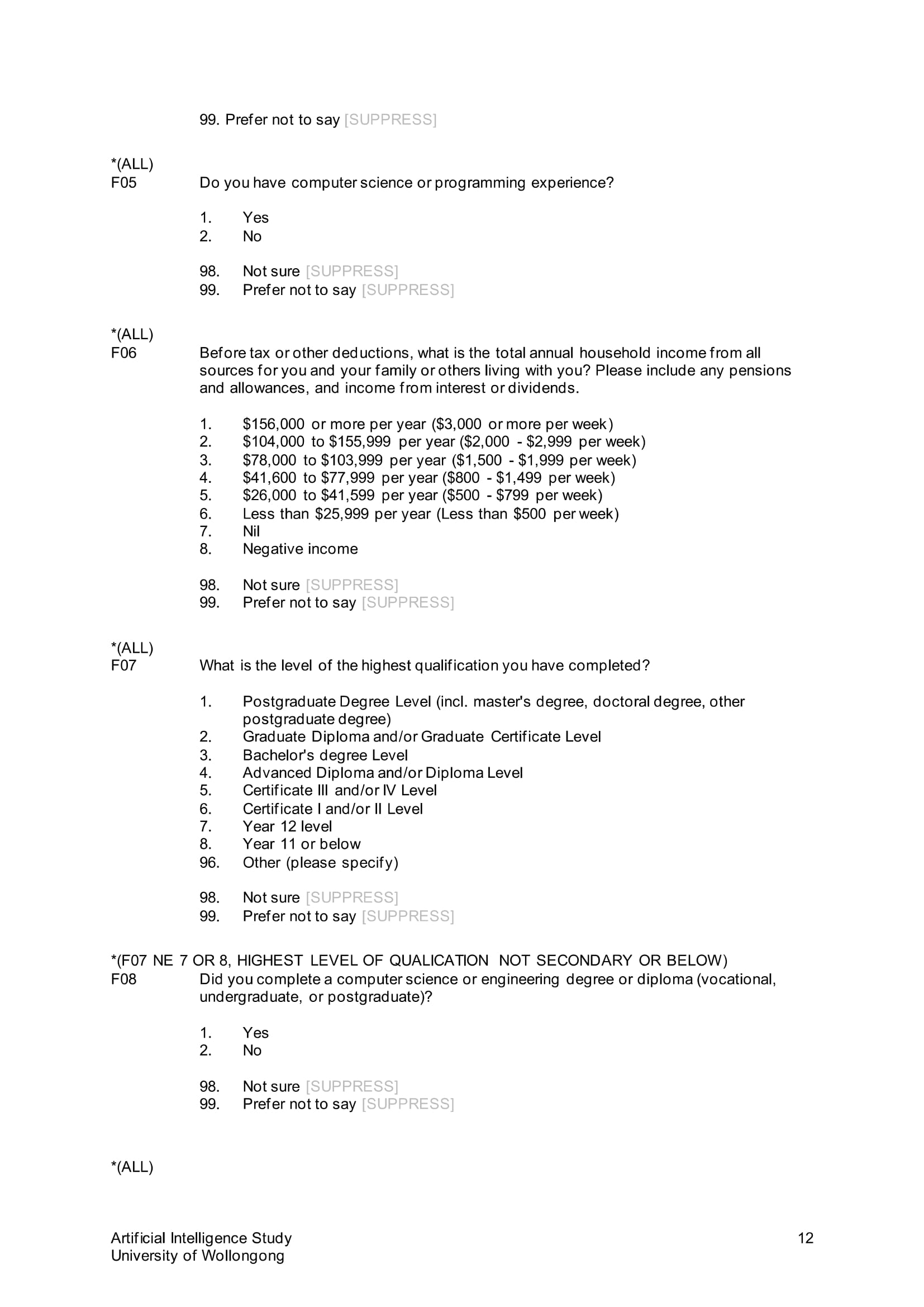

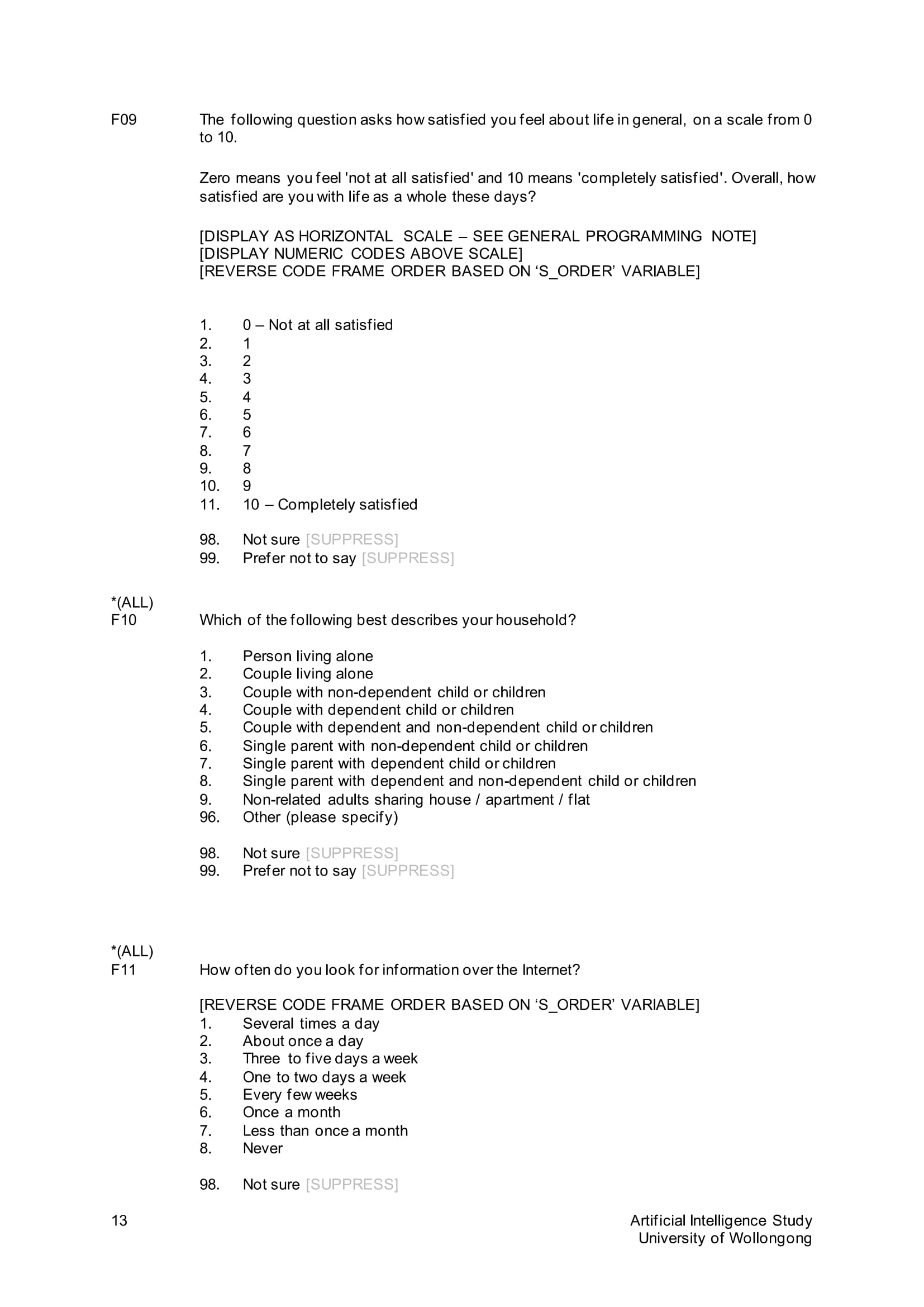

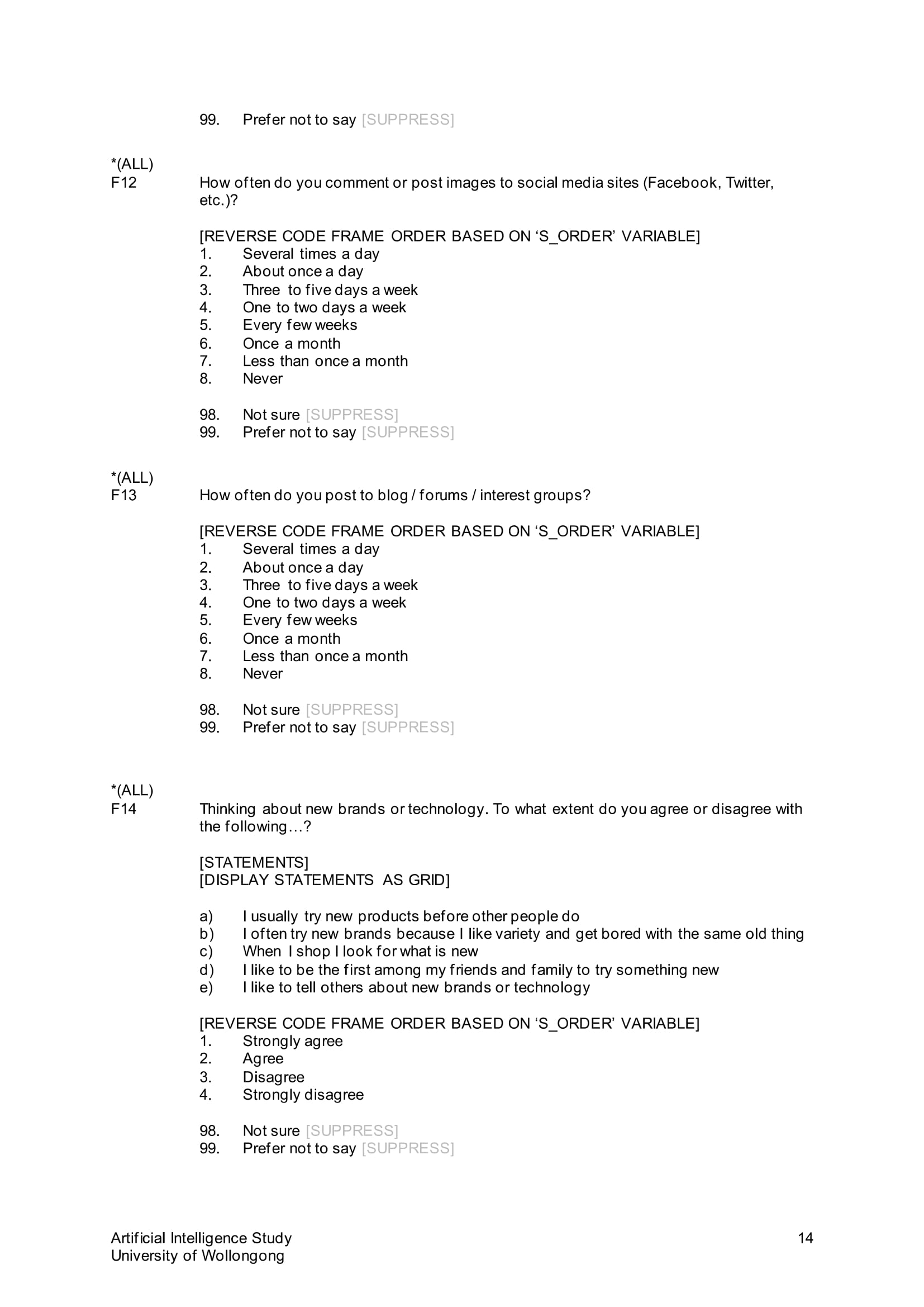

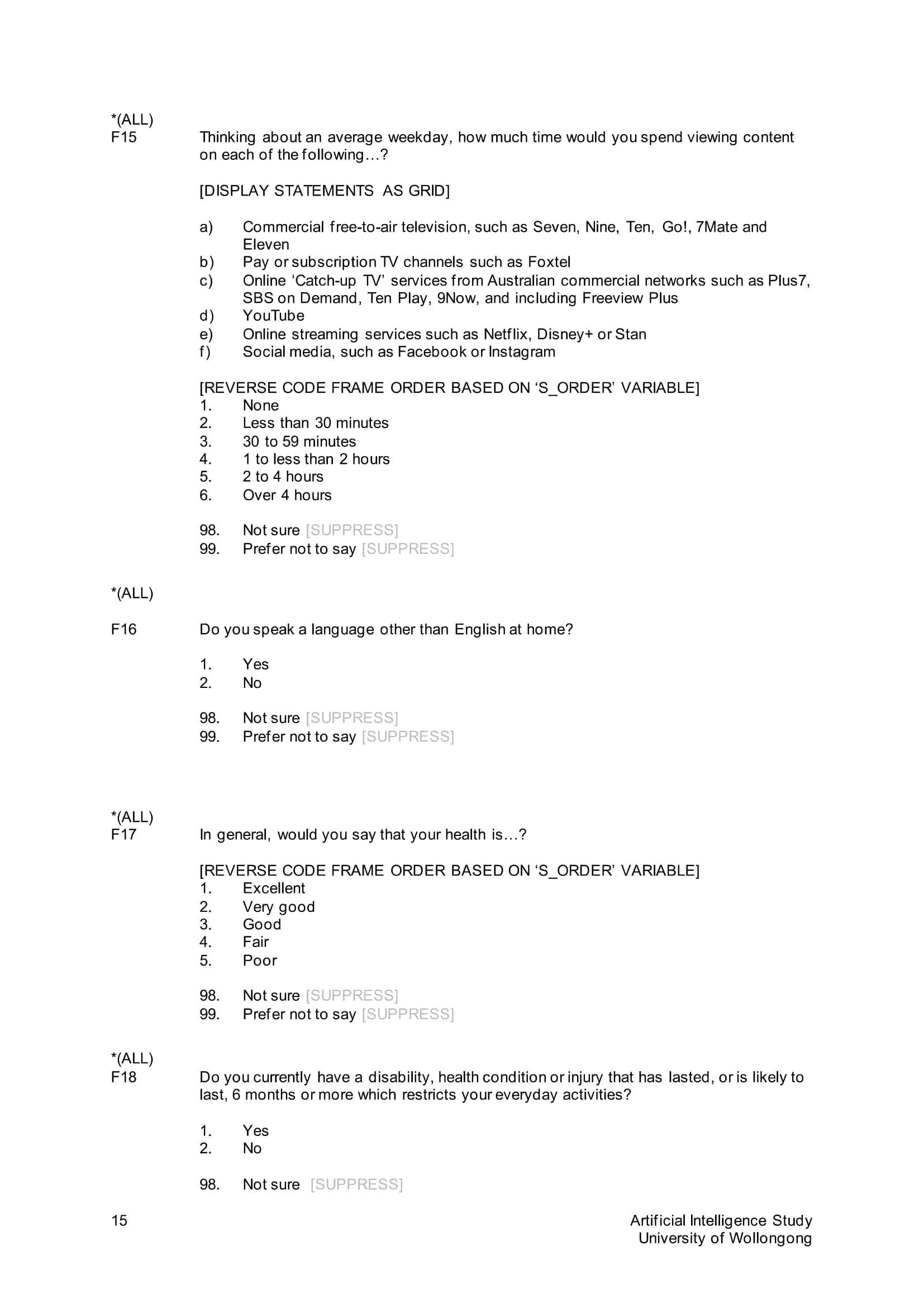

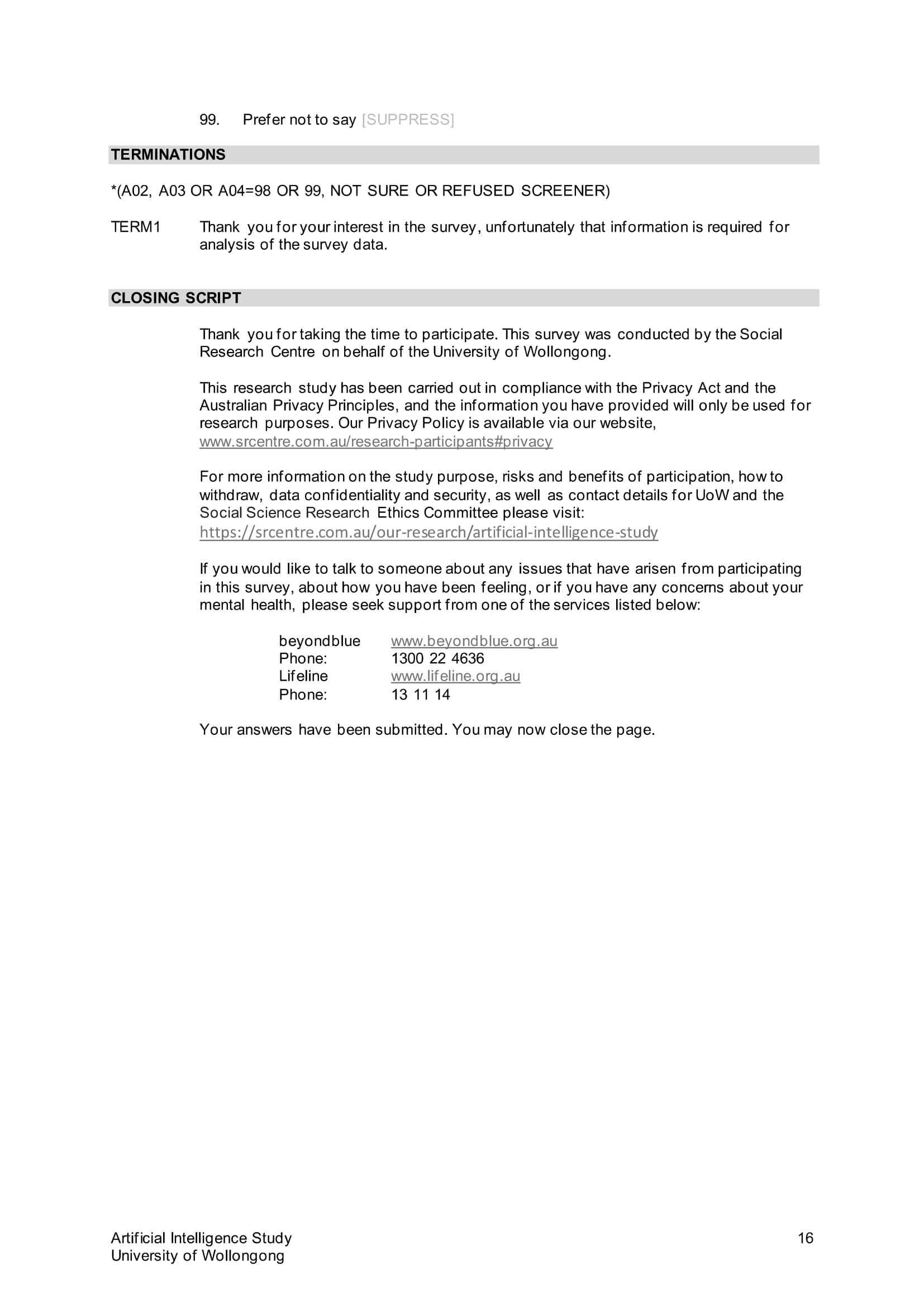
**

Supplement: Multimedia Appendix 1 [file jmir_v24i8e37611_app1.docx]
