# Supplementary material for: The Adoption of Artificial Intelligence in Health Care and Social Services in Australia: Findings From a Methodologically Innovative National Survey of Values and Attitudes (the AVA-AI Study)
Source: J Med Internet Res. 2022 Aug 22;24(8):e37611. doi: 10.2196/37611 (PMC9446139; doi:10.2196/37611)
Supplement: Multimedia Appendix 2 [file jmir_v24i8e37611_app2.docx]

**Table S1. Extended sample composition**

|  | Combined Sample | | | | Online Panel | | | |
| --- | --- | --- | --- | --- | --- | --- | --- | --- |
|  | **Frequency** | | **Percentage** | | **Frequency** | | **Percentage** | |
|  | UW | W | UW | W | UW | W | UW | W |
| *State/Territory* |  |  |  |  |  |  |  |  |
| NSW | 1360 | 1423 | 30.6% | 32.0% | 645 | 635 | 32.3% | 32.5% |
| VIC | 1101 | 1166 | 24.8% | 26.2% | 499 | 497 | 25.0% | 25.5% |
| QLD | 938 | 882 | 21.1% | 19.8% | 404 | 391 | 20.2% | 20.0% |
| SA | 374 | 313 | 8.4% | 7.0% | 150 | 130 | 7.5% | 6.7% |
| WA | 430 | 505 | 9.7% | 11.4% | 208 | 216 | 10.4% | 11.1% |
| TAS | 131 | 79 | 2.9% | 1.8% | 47 | 33 | 2.4% | 1.7% |
| NT | 23 | 13 | 0.5% | 0.3% | 13 | 10 | 0.7% | 0.5% |
| ACT | 91 | 67 | 2.0% | 1.5% | 34 | 39 | 1.7% | 2.0% |
| Not stated/Unknown | 0 | 0 | 0 | 0 | 0 | 0 | 0.0 | 0 |
| *Part of State* |  |  |  |  |  |  |  |  |
| Capital City | 2747 | 2957 | 61.8% | 66.5% | 1338 | 1300 | 66.9% | 66.7% |
| Rest of State | 1693 | 1481 | 38.1% | 33.3% | 654 | 640 | 32.7% | 32.8% |
| Not stated/Unknown | 8 | 10 | 0.2% | 0.2% | 8 | 10 | 0.4% | 0.5% |
| *Age Group* |  |  |  |  |  |  |  |  |
| 18- 24 | 343 | 536 | 7.7% | 12.0% | 242 | 275 | 12.1% | 14.1% |
| 25 - 34 | 697 | 850 | 15.7% | 19.1% | 382 | 362 | 19.1% | 18.6% |
| 35 - 44 | 709 | 750 | 15.9% | 16.9% | 343 | 334 | 17.2% | 17.1% |
| 45 - 54 | 713 | 722 | 16.0% | 16.2% | 326 | 326 | 16.3% | 16.7% |
| 55 - 64 | 783 | 655 | 17.6% | 14.7% | 296 | 285 | 14.8% | 14.6% |
| 65 - 74 | 736 | 511 | 16.5% | 11.5% | 232 | 212 | 11.6% | 10.9% |
| 75 or more | 444 | 394 | 10.0% | 8.9% | 179 | 156 | 9.0% | 8.0% |
| Not stated/Unknown | 23 | 30 | 0.5% | 0.7% | 0 | 0 | 0% | 0% |
| *Gender* |  |  |  |  |  |  |  |  |
| Male | 2082 | 2180 | 46.8% | 49.0% | 989 | 939 | 49.5% | 48.1% |
| Female | 2358 | 2259 | 53.0% | 50.8% | 1010 | 1011 | 50.5% | 51.8% |
| Other | 8 | 9 | 0.2% | 0.2% | 1 | 1 | 0.1% | 0.1% |
| Not stated/Unknown | 0 | 0 | 0.0% | 0.0% | 0 | 0 | 0.0% | 0.0% |
| *Employment Status* |  |  |  |  |  |  |  |  |
| Employed | 2330 | 2709 | 52.4% | 60.9% | 872 | 1061 | 43.6% | 54.4% |
| Not employed | 2112 | 1735 | 47.5% | 39.0% | 1128 | 890 | 56.4% | 45.6% |
| Not stated/Unknown | 6 | 4 | 0.1% | 0.1% | 0 | 0 | 0.0% | 0.0% |
| *Highest Education* |  |  |  |  |  |  |  |  |
| Postgraduate Degree | 533 | 364 | 12.0% | 8.2% | 197 | 162 | 9.9% | 8.3% |
| Graduate Diploma or Graduate Certificate | 309 | 165 | 6.9% | 3.7% | 105 | 84 | 5.3% | 4.3% |
| Bachelor’s degree | 977 | 694 | 22.0% | 15.6% | 456 | 373 | 22.8% | 19.1% |
| Advanced Diploma or Diploma | 542 | 699 | 12.2% | 15.7% | 259 | 303 | 13.0% | 15.5% |
| Certificate III or IV | 703 | 870 | 15.8% | 19.6% | 317 | 343 | 15.9% | 17.6% |
| Certificate I or II | 66 | 67 | 1.5% | 1.5% | 56 | 55 | 2.8% | 2.8% |
| Year 12 | 625 | 910 | 14.1% | 20.5% | 325 | 370 | 16.3% | 19.0% |
| Years 11 and below | 594 | 582 | 13.4% | 13.1% | 279 | 256 | 14.0% | 13.1% |
| Not stated/Unknown | 99 | 96 | 2.2% | 2.2% | 6 | 5 | 0.3% | 0.3% |
| *Gross weekly household income* | | | | |  |  |  |  |
| $3,000 or more a week | 604 | 635 | 13.6% | 14.3% | 176 | 211 | 8.8% | 10.8% |
| $2,000 - $2,999 a week | 637 | 660 | 14.3% | 14.8% | 283 | 295 | 14.2% | 15.1% |
| $1,500 - $1,999 a week | 647 | 621 | 14.5% | 14.0% | 333 | 294 | 16.7% | 15.1% |
| $800 - $1,499 a week | 998 | 1038 | 22.4% | 23.3% | 496 | 497 | 24.8% | 25.5% |
| $500 - $799 a week | 649 | 608 | 14.6% | 13.7% | 331 | 296 | 16.6% | 15.2% |
| Less than $500 a week | 591 | 550 | 13.3% | 12.4% | 281 | 261 | 14.1% | 13.4% |
| Nil | 115 | 139 | 2.6% | 3.1% | 67 | 70 | 3.4% | 3.6% |
| Negative income | 45 | 34 | 1.0% | 0.8% | 33 | 26 | 1.7% | 1.3% |
| Not stated/Unknown | 162 | 162 | 3.6% | 3.6% | 0 | 0 | 0.0% | 0.0% |
| *Other language at home* | | |  |  |  |  |  |  |
| Yes | 640 | 1036 | 14.4% | 23.3% | 315 | 438 | 15.8% | 22.5% |
| No | 3807 | 3411 | 85.6% | 76.7% | 1685 | 1513 | 84.3% | 77.5% |
| Not stated/Unknown | 1 | 1 | 0.02% | 0.02% | 0 | 0 | 0.0% | 0.0% |
| *General Health* |  |  |  |  |  |  |  |  |
| Excellent | 416 | 549 | 9.4% | 12.3% | 186 | 236 | 9.3% | 12.1% |
| Very good | 1431 | 1887 | 32.2% | 42.4% | 619 | 837 | 31.0% | 42.9% |
| Good | 1627 | 1302 | 36.6% | 29.3% | 727 | 562 | 36.4% | 28.8% |
| Fair | 774 | 573 | 17.4% | 12.9% | 372 | 255 | 18.6% | 13.1% |
| Poor | 191 | 131 | 4.3% | 2.9% | 96 | 59 | 4.8% | 3.0% |
| Not stated/Unknown | 9 | 6 | 0.2% | 0.1% | 0 | 0 | 0,0% | 0.0% |
| *Internet usage* |  |  |  |  |  |  |  |  |
| Several times a day | 2563 | 2776 | 57.6% | 62.4% | 1061 | 1144 | 53.1% | 58.6% |
| About once a day | 829 | 745 | 18.6% | 16.8% | 402 | 349 | 20.1% | 17.9% |
| Three to five days a week | 383 | 340 | 8.6% | 7.6% | 198 | 168 | 9.9% | 8.6% |
| One to two days a week | 311 | 277 | 7.0% | 6.2% | 150 | 129 | 7.5% | 6.6% |
| Every few weeks | 145 | 120 | 3.3% | 2.7% | 88 | 68 | 4.4% | 3.5% |
| Once a month | 74 | 68 | 1.7% | 1.5% | 37 | 35 | 1.9% | 1.8% |
| Less than once a month | 76 | 70 | 1.7% | 1.6% | 52 | 47 | 2.6% | 2.4% |
| Never | 66 | 51 | 1.5% | 1.1% | 11 | 10 | 0.6% | 0.5% |
| Not stated/Unknown | 1 | 1 | 0.2% | 0.2% | 1 | 1 | 0.1% | 0.1% |
