# Supplementary material for: The Adoption of Artificial Intelligence in Health Care and Social Services in Australia: Findings From a Methodologically Innovative National Survey of Values and Attitudes (the AVA-AI Study)
Source: J Med Internet Res. 2022 Aug 22;24(8):e37611. doi: 10.2196/37611 (PMC9446139; doi:10.2196/37611)
Supplement: Multimedia Appendix 3 [file jmir_v24i8e37611_app3.docx]

| Table S2. Support/oppose specific AI scenarios | | | | | | | | | | | | | | | |
| --- | --- | --- | --- | --- | --- | --- | --- | --- | --- | --- | --- | --- | --- | --- | --- |
| Support/oppose specific AI scenarios | | | | | | | | | | | | | | | |
| *Non-explainable hospital algorithms (C03)* | | | | | | | *Targeted compliance checking (D03)* | | | | | | | | |
| *Data sharing for quality care (C04)* | | | | | | | *Non-explainable job services (D04)* | | | | | | | | |
| *De-skilling doctors (C05)* | | | | | | |  | | | | | | | | |
|  | **1 - I support this use of AI** | | | **2** | | | **3** | | | **4** | | | **5 - I oppose this use of AI** | | |
|  | **Est.** | **95% CI** | | **Est.** | **95% CI** | | **Est.** | **95% CI** | | **Est.** | **95% CI** | | **Est.** | **95% CI** | |
|  |  | Lower | Upper |  | Lower | Upper |  | Lower | Upper |  | Lower | Upper |  | Lower | Upper |
| Non-explainable hospital algorithms (C03) | 10.6% | 9.1% | 12.3% | 18.5% | 16.4% | 20.7% | 29.3% | 26.8% | 32.0% | 18.4% | 16.4% | 20.5% | 23.2% | 20.9% | 25.7% |
| Data sharing for quality care (C04) | 14.8% | 13.0% | 16.8% | 27.5% | 25.1% | 30.1% | 26.8% | 24.5% | 29.3% | 14.2% | 12.3% | 16.2% | 16.7% | 14.6% | 18.9% |
| De-skilling doctors (C05) | 10.4% | 8.9% | 12.3% | 16.5% | 14.6% | 18.7% | 24.7% | 22.4% | 27.1% | 20.7% | 18.5% | 23.1% | 27.6% | 25.2% | 30.2% |
| Targeted compliance checking (D03) | 15.7% | 13.8% | 17.9% | 23.2% | 21.0% | 25.6% | 28.1% | 25.7% | 30.7% | 13.1% | 11.4% | 15.0% | 19.9% | 17.7% | 22.2% |
| Non-explainable job services (D04) | 10.1% | 8.7% | 11.8% | 21.1% | 18.9% | 23.4% | 29.2% | 26.8% | 31.8% | 19.2% | 17.0% | 21.5% | 20.4% | 18.2% | 22.8% |
| Automated parent support with limited contestability (D05) | 11.9% | 10.3% | 13.7% | 23.0% | 20.7% | 25.5% | 30.7% | 28.2% | 33.3% | 16.0% | 14.1% | 18.1% | 18.4% | 16.3% | 20.7% |
