# Supplementary material for: The Adoption of Artificial Intelligence in Health Care and Social Services in Australia: Findings From a Methodologically Innovative National Survey of Values and Attitudes (the AVA-AI Study)
Source: J Med Internet Res. 2022 Aug 22;24(8):e37611. doi: 10.2196/37611 (PMC9446139; doi:10.2196/37611)
Supplement: Multimedia Appendix 4 [file jmir_v24i8e37611_app4.docx]

| Table S3. Importance of health (C) and welfare (D) scenarios | | | | | | | | | | | | | | | |
| --- | --- | --- | --- | --- | --- | --- | --- | --- | --- | --- | --- | --- | --- | --- | --- |
| Estimated Proportions and 95 percent Confidence intervals for importance of AI attributes | | | | | | | | | | | | | | | |
| *C01 – Machine reads medical test, diagnoses, recommends treatment* | | | | | | | *D01 – Machine processes application for unemployment benefits (data sharing required)* | | | | | | | | |
| *C02 – Machine triages when you are unwell* | | | | | | | *D02 – Chatbot advises about carers payments* | | | | | | | | |
|  | **Extremely important** | | | **Very important** | | | **Important** | | | **Slightly important** | | | **Not at all important** | | |
|  | **Est.** | **95% CI** | | **Est.** | **95% CI** | | **Est.** | **95% CI** | | **Est.** | **95% CI** | | **Est.** | **95% CI** | |
|  |  | Lower | Upper |  | Lower | Upper |  | Lower | Upper |  | Lower | Upper |  | Lower | Upper |
| Explanation (C01) | 44.1% | 42.1% | 46.0% | 32.0% | 30.2% | 33.9% | 18.6% | 17.1% | 20.2% | 4.6% | 3.7% | 5.6% | 0.7% | 0.4% | 1.2% |
| Speed (C01) | 33.6% | 31.7% | 35.5% | 32.2% | 30.4% | 34.1% | 27.7% | 26.0% | 29.6% | 5.1% | 4.3% | 6.1% | 1.3% | 0.9% | 1.9% |
| Accuracy (C01) | 66.8% | 64.9% | 68.7% | 20.8% | 19.2% | 22.4% | 9.2% | 8.2% | 10.4% | 2.4% | 1.8% | 3.3% | 0.7% | 0.5% | 1.2% |
| Human contact (C01) | 50.3% | 48.3% | 52.3% | 27.6% | 25.9% | 29.4% | 17.1% | 15.6% | 18.7% | 4.2% | 3.4% | 5.2% | 0.8% | 0.5% | 1.3% |
| Responsibility (C01) | 48.8% | 46.8% | 50.8% | 29.6% | 27.9% | 31.5% | 16.7% | 15.3% | 18.3% | 3.9% | 3.1% | 4.9% | 0.9% | 0.6% | 1.4% |
| Reducing costs (C01) | 29.1% | 27.3% | 30.9% | 28.8% | 27.0% | 30.6% | 28.8% | 27.0% | 30.6% | 10.0% | 8.8% | 11.4% | 3.3% | 2.6% | 4.1% |
| Fairness (C01) | 44.4% | 42.5% | 46.4% | 29.6% | 27.8% | 31.4% | 21.4% | 19.8% | 23.1% | 3.5% | 2.8% | 4.4% | 1.1% | 0.8% | 1.6% |
| Explanation (C02) | 43.7% | 41.0% | 46.5% | 31.6% | 29.0% | 34.2% | 20.0% | 17.9% | 22.3% | 3.2% | 2.3% | 4.6% | 1.5% | 0.9% | 2.5% |
| Speed (C02) | 41.2% | 38.5% | 44.0% | 32.6% | 30.1% | 35.3% | 22.0% | 19.7% | 24.4% | 3.1% | 2.3% | 4.2% | 1.1% | 0.6% | 1.9% |
| Accuracy (C02) | 62.8% | 60.0% | 65.4% | 22.3% | 20.0% | 24.7% | 12.4% | 10.7% | 14.3% | 1.4% | 0.9% | 2.2% | 1.2% | 0.6% | 2.2% |
| Human contact (C02) | 49.0% | 46.2% | 51.8% | 26.9% | 24.5% | 29.4% | 19.7% | 17.6% | 22.1% | 3.1% | 2.3% | 4.3% | 1.2% | 0.7% | 2.3% |
| Responsibility (C02) | 49.7% | 46.9% | 52.5% | 29.6% | 27.2% | 32.3% | 16.7% | 14.8% | 18.9% | 2.6% | 1.8% | 3.8% | 1.3% | 0.7% | 2.3% |
| Reducing costs (C02) | 25.5% | 23.2% | 27.9% | 27.2% | 24.8% | 29.7% | 32.0% | 29.4% | 34.6% | 9.5% | 8.0% | 11.2% | 5.9% | 4.5% | 7.6% |
| Fairness (C02) | 42.4% | 39.7% | 45.1% | 29.5% | 27.1% | 32.1% | 22.1% | 19.8% | 24.5% | 3.8% | 2.8% | 5.1% | 2.2% | 1.4% | 3.5% |
| Explanation (D01) | 45.0% | 42.3% | 47.8% | 30.0% | 27.5% | 32.6% | 20.6% | 18.5% | 22.9% | 3.2% | 2.3% | 4.3% | 1.2% | 0.7% | 2.0% |
| Speed (D01) | 36.3% | 33.7% | 39.0% | 34.8% | 32.2% | 37.6% | 23.8% | 21.5% | 26.3% | 4.0% | 3.1% | 5.2% | 1.0% | 0.6% | 1.8% |
| Accuracy (D01) | 58.3% | 55.6% | 61.0% | 25.8% | 23.5% | 28.3% | 13.3% | 11.6% | 15.3% | 1.6% | 1.2% | 2.3% | 0.9% | 0.5% | 1.6% |
| Human contact (D01) | 44.9% | 42.1% | 47.6% | 29.6% | 27.1% | 32.3% | 19.8% | 17.8% | 22.0% | 4.5% | 3.5% | 5.8% | 1.2% | 0.7% | 2.0% |
| Reducing costs (D01) | 22.6% | 20.4% | 24.9% | 25.2% | 22.9% | 27.6% | 36.3% | 33.7% | 39.1% | 10.0% | 8.4% | 11.8% | 5.9% | 4.7% | 7.4% |
| Fairness (D01) | 48.1% | 45.3% | 50.8% | 29.1% | 26.6% | 31.7% | 19.3% | 17.2% | 21.6% | 2.4% | 1.8% | 3.3% | 1.2% | 0.7% | 1.9% |
| Personal tailoring (D01) | 43.7% | 40.9% | 46.5% | 31.6% | 29.1% | 34.2% | 20.3% | 18.2% | 22.6% | 3.1% | 2.4% | 4.2% | 1.3% | 0.8% | 2.0% |
| Explanation (D02) | 37.8% | 35.2% | 40.6% | 31.7% | 29.1% | 34.3% | 23.3% | 21.0% | 25.7% | 5.4% | 4.3% | 6.8% | 1.8% | 1.1% | 3.0% |
| Speed (D02) | 39.5% | 36.9% | 42.3% | 34.7% | 32.1% | 37.4% | 21.7% | 19.5% | 24.2% | 2.8% | 2.0% | 4.0% | 1.2% | 0.6% | 2.1% |
| Accuracy (D02) | 59.8% | 57.0% | 62.4% | 24.2% | 21.9% | 26.6% | 13.5% | 11.7% | 15.5% | 1.7% | 1.2% | 2.4% | 0.8% | 0.4% | 1.6% |
| Human contact (D02) | 48.1% | 45.3% | 50.9% | 27.5% | 25.0% | 30.1% | 19.4% | 17.3% | 21.6% | 3.7% | 2.8% | 5.0% | 1.3% | 0.8% | 2.2% |
| Reducing costs (D02) | 21.9% | 19.7% | 24.1% | 24.0% | 21.8% | 26.4% | 33.6% | 31.0% | 36.2% | 13.1% | 11.2% | 15.2% | 7.5% | 6.1% | 9.3% |
| Fairness (D02) | 46.7% | 43.9% | 49.5% | 29.6% | 27.1% | 32.2% | 20.5% | 18.4% | 22.9% | 2.1% | 1.4% | 3.2% | 1.1% | 0.6% | 2.0% |
| Personal tailoring (D02) | 46.2% | 43.4% | 49.0% | 31.0% | 28.5% | 33.6% | 18.7% | 16.6% | 21.0% | 3.0% | 2.2% | 4.2% | 1.1% | 0.6% | 2.0% |
